# Heterogeneity in the definition of delirium in ICUs and association with the intervention effect in randomized controlled trials: a meta-epidemiological study

**DOI:** 10.1186/s13054-023-04411-y

**Published:** 2023-05-04

**Authors:** Lucie Collet, Aymeric Lanore, Camille Alaterre, Jean-Michel Constantin, Guillaume L. Martin, Agnès Caille, Arthur James, Agnès Dechartres

**Affiliations:** 1 Sorbonne Université, INSERM, Institut Pierre Louis d’Épidémiologie et de Santé Publique, AP-HP, Hôpital Pitié-Salpetrière, Département de Santé Publique, 47-83 Boulevard de l’Hôpital, 75013 Paris, France; 2 Sorbonne Université, GRC 29, DMU DREAM, AP-HP, Hôpital Pitié-Salpetrière, Département d’Anesthésie-Réanimation, Paris, France; 3 CIC Neurosciences, Department of Neurology, Assistance Publique Hôpitaux de Paris, Hôpital Pitié-Salpêtrière, Paris, France; 4 Université́ de Tours, Université́ de Nantes, INSERM, SPHERE U1246, Tours, France; INSERM CIC 1415, CHRU de Tours, Tours, France

**Keywords:** Meta-analysis, Meta-epidemiology, Delirium, Heterogeneity, Definition, Intensive care

## Abstract

**Purpose:**

To evaluate the heterogeneity in the definition of delirium in randomized controlled trials (RCTs) included in meta-analyses of delirium in intensive care units (ICUs) and to explore whether intervention effect depends on the definition used.

**Methods:**

We searched PubMed for meta-analyses including RCTs evaluating prevention or treatment strategies of delirium in ICU. The definition of delirium was collected from RCTs and classified as validated (DSM criteria, CAM-ICU, ICDSC, NEECHAM, DRS-R98) or non-validated (non-validated scales, set of symptoms, physician appreciation or not reported). We conducted a meta-epidemiological analysis to compare intervention effects between trials using or not a validated definition by a two-step method as primary analysis and a multilevel model as secondary analysis. A ratio of odds ratios (ROR) < 1 indicated larger intervention effects in trials using a non-validated definition.

**Results:**

Of 149 RCTs (41 meta-analyses), 109 (73.1%) used a validated definition and 40 (26.8%) did not (including 31 [20.8%] not reporting the definition). The primary analysis of 7 meta-analyses (30 RCTs) found no significant difference in intervention effects between trials using a validated definition and the others (ROR = 0.54, 95% CI 0.27–1.08), whereas the secondary multilevel analysis including 12 meta-analyses (67 RCTs) found significantly larger effects for trials using a non-validated versus a validated definition (ROR = 0.36, 95% CI 0.21–0.62).

**Conclusion:**

The definition of delirium was heterogeneous across RCTs, with one-fifth not reporting how they evaluated delirium. We did not find a significant association with intervention effect in the primary analysis. The secondary analysis including more studies revealed significantly larger intervention effects in trials using a non-validated versus a validated definition.

**Supplementary Information:**

The online version contains supplementary material available at 10.1186/s13054-023-04411-y.

## Introduction

Delirium is a neuropsychiatric syndrome reflecting an acute brain dysfunction that occurs frequently in intensive care units (ICUs) [1]. It may be induced by a physiological stress related to a systemic pathology or to the critical care interventions and other specific factors (e.g., sleep disturbances, light pollution at night). Characteristics such as age, sex, disease severity, mechanical ventilation are risk factors [1, 2]. The incidence is about 30%, with significant variations (from 10 to 80%) depending on the admission cause and the definition used to characterize it [3]. This disorder is challenging to assess reliably because of varied symptomatology, including fluctuating mental status, disturbance in consciousness, attention and judgment disorders, disorientation, circadian disturbances, and psychomotor slowing and/or agitation, which could lead to under-recognition [4, 5]. The reference diagnosis is based on the diagnostic and statistical manual of mental disorders (DSM) criteria after a psychiatric evaluation. To facilitate the diagnosis and recognition of this trouble by ICU physicians in their everyday practice, scales were developed in the 2000s and included the Confusion Assessment Method for the Intensive Care Unit (CAM-ICU) or the Intensive Care Delirium Screening Checklist (ICDSC). Both scales were validated after comparison with the DSM criteria [6].

Delirium is associated with increased mortality, prolonged hospital stay, prolonged mechanical ventilation, and increased risk of long-term cognitive impairment [7–10]; therefore, it is a major therapeutic issue. Current research on delirium in ICUs focuses on the evaluation of prevention and treatment strategies including various pharmacological or non-pharmacological interventions that have been evaluated in many randomized controlled trials (RCTs) [11]. However, few studies have raised the issue of the definition of delirium, which seems to be heterogeneous in the published literature despite the existing tools. The definition of an outcome such as delirium may be an important source of heterogeneity and variation in the intervention effect making comparison between trials on this topic difficult [12].

In this study, we aimed to evaluate the heterogeneity in the definition of delirium used in RCTs included in meta-analyses evaluating the prevention or treatment of delirium in ICUs and to explore whether the intervention effect varies depending on the definition used.

## Methods

### Study design

Our study used a meta-epidemiological approach, the reference method for identifying biases in RCTs [13]. This method consists of assessing whether a given characteristic is associated with the intervention effect in a sample of meta-analyses [14]. First, a systematic review was conducted to identify meta-analyses assessing prevention or treatment strategies of delirium in ICUs. This systematic review followed the Preferred Reporting Items for Systematic Reviews and Meta-analyses (PRISMA) (Additional file 1: Table S1). Then, we evaluated the definition of delirium in each included trial report and classified the definition as validated and not validated. Finally, we compared intervention effects between trials reporting a validated definition and those that did not by using meta-epidemiological analyses.

### Search strategy

We identified systematic reviews with meta-analyses evaluating prevention or treatment strategies of delirium in ICUs by searching PubMed and the Cochrane Database of Systematic Reviews on March, 4, 2022. The detailed search equation is reported in Additional file 1: Information S1. We also manually searched the “Emergency and Critical care” and “Dementia and Cognitive improvement” review groups of Cochrane.

### Selection of relevant meta-analyses

Two reviewers (LC and CA) independently assessed the eligibility of retrieved references after removing duplicates. Discrepancies were resolved by discussion with a third reviewer (AD) to reach consensus.

Eligibility criteria were meta-analyses including RCTs of adults hospitalized in medical or surgical ICUs that assessed an intervention for preventing or treating delirium and evaluating delirium as a primary or secondary outcome. We included network meta-analyses if direct comparisons were available and focused on these. If a systematic review included several comparisons evaluating different types of interventions, we included each comparison corresponding to our eligibility criteria.

We excluded meta-analyses dedicated to neurobehavioral manifestations in neurological patients or to delirium in alcohol withdrawal, systematic reviews without meta-analysis, protocols and meta-epidemiological studies or overviews. We also excluded meta-analyses including fewer than three RCTs because three is the minimum to conduct meta-epidemiological analyses.

### Selection of RCTs within meta-analyses

For each selected meta-analysis of delirium, we included only RCTs and excluded RCTs of children and those not conducted in an ICU. We then removed duplicates. We did not consider as duplicates the same RCTs if, across meta-analyses, a different definition of delirium was used or if the RCT was conducted in different populations or if the experimental or control intervention were different. For example, a three-arm RCT could be included twice if a meta-analysis considered the comparison of arms A and B and another considered the comparison of arms A and C. An RCT evaluating delirium with different definitions could also be included twice if a meta-analysis considered the incidence of delirium based on one definition and another considered the incidence of delirium based on the second definition.

### Data extraction

Two reviewers (LC and CA/AL) independently extracted data; any disagreements were resolved by discussion with a third reviewer (AD). Two data collection forms were developed and used: one for meta-analyses and one for individual trials.

For each meta-analysis, the following data were extracted:

- General characteristics: date of publication, journal, funding sources
- Number of studies included in the meta-analysis of delirium
- Interventions assessed in the experimental and control groups
- Delirium outcome evaluated: incidence, duration, delirium- or coma-free days, severity
- Tool used to assess risk of bias
- Method for pooling data
- Results of the meta-analysis of delirium: combined estimate with confidence intervals (CIs) and heterogeneity assessed with the *I*^2^ and Cochran *Q* chi-square test.
- Whether and how review authors discussed the definition of delirium in included studies

For each trial included in the meta-analysis of delirium, we collected:

- General characteristics: date of publication, journal, funding sources, reporting of registration in a clinical trial registry (e.g., ClinicalTrials.gov)
- Population characteristics: sample size, main inclusion and exclusion criteria
- Details on the experimental and control interventions
- Primary outcome defined in the trial
- Assessment of risk of bias using the Cochrane Risk of Bias 1 (RoB1) tool [15]: random sequence generation, allocation concealment, blinding of participants and personnel, blinding of outcome assessors, incomplete outcome data, selective reporting
- Definition of delirium used: DSM criteria, scale (CAM-ICU, ICDSC, NEECHAM, other), set of symptoms, physician appreciation, other, not reported. If an RCT did not report delirium as an outcome but the authors of the meta-analysis interpreted an outcome as delirium, we considered the definition as not reported.
- Results:
  - Number of events and analyzed patients in each group for the incidence of delirium
  - Mean, standard deviation, and number of analyzed patients in each group for the duration of delirium and number of delirium- and coma-free days. When the mean and standard deviation were not available, we extracted the median and interquartile range and converted them to mean and standard deviation [16].

Data for RCTs were extracted directly from the RCT report. If the full text was not available, we contacted the authors, and in case of no answer, we collected the data from the meta-analysis. The risk of bias was extracted from meta-analyses. Because meta-analyses used different tools, we relied on the Cochrane RoB1 tool because it is a reference tool and was the most frequently used. We re-evaluated the risk of bias from the RCT report by using this tool when another tool was used in the meta-analysis.

### Definition of delirium in included RCTs

We evaluated whether the authors used a validated definition or not based on the literature including a list of assessment tools to measure delirium with COSMIN ratings published by the Network for Investigations of Delirium: Unifying Scientists (NIDUS) [17]. We considered as validated definitions the DSM criteria as this is the gold standard and tools that had been compared and validated against the DSM criteria (CAM-ICU, ICDSC, NEECHAM and Delirium rating scale Revised-98 (DRS-R98)) [6, 18–22]. Non-validated definitions were non-validated scales (RASS or not reported), a set of symptoms, physician appreciation or the definition not reported.

### Data synthesis

#### Meta-analyses

We estimated the intervention effect for the incidence of delirium with odds ratios (ORs) calculated from the number of patients presenting the outcome and the number of patients analyzed in the experimental and control groups. Outcome events were re-coded so that an OR < 1 indicated a beneficial effect of the experimental intervention. Concerning delirium duration, number of delirium- or coma-free days, we estimated a standardized mean difference by dividing the difference in means between groups by the standard deviation among participants. DerSimonian and Laird random-effects models were used to combine intervention effects across RCTs within each meta-analysis. Heterogeneity across trials was assessed by the *I*^2^ and the Cochran *Q* Chi-square test.

#### Meta-epidemiological analyses

For these analyses, we focused on meta-analyses of the incidence of delirium including trials comparing an intervention to a placebo or usual care. We excluded meta-analyses comparing two active interventions because the direction of bias may be uncertain in that case. We also excluded meta-analyses evaluating the same research question (same intervention and control group) if they had three or more RCTs in common.

##### Primary analysis

The primary meta-epidemiological analysis followed the two-step method described by Sterne et al. [23]. We compared intervention effects between RCTs using a validated definition and those using a non-validated one as follows. For each meta-analysis, we first estimated the ratio of ORs (ROR) defined as the OR from trials reporting a non-validated definition to the OR from those reporting a validated definition by using a random-effects meta-regression model to incorporate between-trial heterogeneity. An ROR < 1 indicated larger intervention effect estimates in trials using a non-validated versus a validated definition. Then, we estimated the combined ROR and its 95% CI by using a random-effects meta-analysis model. Heterogeneity across RORs was assessed with the *I*^2^, the Cochran *Q* Chi-square test, and the between-meta-analysis variance τ^2^.

***Subgroup and sensitivity analyses for the primary analysis*** We conducted subgroup analysis by type of intervention assessed (pharmacological or non-pharmacological) and tested interaction with a random-effects meta-regression model.

To control for potential confounders, we conducted sensitivity analyses by adjusting the meta-regression model for each item of the RoB1 tool, sample size and publication date. The cutoff chosen for publication date was 2010 because the definition of delirium evolved with the development of scales such as CAM-ICU and ICDSC that were more often used after 2010 in ICUs and in the research leading to more screening, prevention and treatment of delirium.

##### Secondary analyses

The secondary meta-epidemiological analyses were conducted using another method, a one-step multilevel logistic regression model with random effects described by Siersma et al. [24]. Two comparisons were conducted: first, we compared intervention effects between trials using a validated definition and those using a non-validated one. Then, we compared four definition categories that we considered the most representative and relevant categories. These four categories were the DSM criteria as the reference category, the CAM-ICU, non-validated scales and definition not reported. Further details on these secondary analyses can be found in Additional file 1: Information S2.

Analyses were performed with R 4.1.3 (R Core Team [2022]. R Foundation for Statistical Computing, Vienna, Austria. https://www.R-project.org) except the multilevel analyses, which were performed with SAS version 9.4 (SAS Institute Inc., Cary, NC, USA).

## Results

### Selection of relevant meta-analyses

The selection process is reported in Additional file 1: Fig. S1. Of 555 references identified, we removed 14 duplicates and selected 41 meta-analyses meeting the eligibility criteria.

### Characteristics of selected meta-analyses

The characteristics of the 41 included meta-analyses are summarized in Table 1 and detailed in Additional file 1: Table S2. Three (7.3%) meta-analyses were from Cochrane. Most (*n* = 30, 73.2%) studied the prevention of delirium. The interventions were mostly pharmacological (*n* = 29, 70.7%). The incidence of delirium was reported as an outcome in 38 (92.7%) meta-analyses, and the duration of delirium was studied in 15 (36.6%). Only seven (17.1%) meta-analyses precisely defined delirium in their selection criteria. The risk of bias was evaluated with the RoB1 tool in 35 (85.4%) meta-analyses. The issue regarding heterogeneity in the definition of delirium was raised in 14 (34.1%), with most highlighting the multiplicity of tools used to define delirium, not all being validated. Five reported that the intervention effect observed could be affected by the multiple ways to define delirium. Two found that the part of the heterogeneity in the incidence of delirium may be due to the different definitions of delirium.

**Table 1:** Main characteristics of the included meta-analyses (*n* = 41)

Characteristics	*N* = 41
Sources, *n* (%)	
Cochrane reviews	3 (7.3)
Non-Cochrane reviews	38 (92.7)
Year of publication, median (*Q*1–*Q*3)	2020 (2016–2021)
At least one author with methodology skills, *n* (%)	11 (26.8)
Funding, *n* (%)	
No specific	18 (43.9)
Public	9 (22.0)
Private	2 (4.9)
Public and private	4 (9.7)
Not reported	8 (19.5)
ICU type, *n* (%)	
Medical and surgical	32 (78.0)
Surgical	1 (2.5)
Medical	0 (0)
Not reported	8 (19.5)
Population type, *n* (%)	
Mechanically ventilated	4 (9.8)
Post-surgery	1 (2.4)
Older patients	1 (2.4)
Other	5 (12.2)
No specific	30 (73.2)
Objective, *n* (%)	
Prevention of delirium	30 (73.2)
Treatment of delirium	5 (12.2)
Both	6 (14.6)
Type of intervention, *n* (%)	
Pharmacological	29 (70.7)
Non-pharmacological	10 (24.4)
Both, *n* (%)	2 (4.9)
Control group*, *n* (%)	
Placebo	21 (51.2)
No treatment	2 (4.9)
Other treatment	24 (58.5)
Usual care	13 (31.7)
Main outcome, *n* (%)	
Incidence of delirium	38 (92.7)
Duration of delirium	15 (36.6)
Severity of delirium	2 (4.9)
Mortality	34 (82.9)
ICU length of stay	35 (85.4)
Hospital length of stay	16 (39.0)
Duration of mechanical ventilation	23 (56.1)
Evaluation of risk of bias, *n* (%)	
RoB 1	35 (85.4)
RoB 2	2 (4.9)
Jadad scale	1 (2.4)
JBI critical appraisal checklist	1 (2.4)
Not evaluated	1 (2.4)
Number of studies included in the meta-analysis of delirium, med (*Q*1–*Q*3)	5 (3–7)
Heterogeneity of delirium raised in the discussion, *n* (%)	14 (34.1)

*ICU* intensive care unit, *RoB1* Cochrane Risk of Bias 1 tool, *RoB2* Cochrane Risk of Bias 2 tool, *JBI* Joanna Briggs Institute

*The total exceeds 41 because in some meta-analyses, different control groups could be used

### Selection and characteristics of RCTs

The 41 meta-analyses included 300 trials; 12 were excluded because the design was not randomized, three because the study was not conducted in ICUs and one because the population studied was pediatric. For the 284 remaining RCTs, we identified 135 duplicates and finally included 149 RCTs (Additional file 1: Fig. S1), mostly published after 2010 (*n* = 111, 74.5%).

Most RCTs studied a pharmacological intervention (*n* = 109, 73.1%) (Table 2), and the main drug was dexmedetomidine in 73 (50.7%) trials. The control group received a placebo in 48 (32.2%) trials, standard care in 36 (24.2%) and another treatment in 61 (40.9%), mostly propofol in 25 (43.1%) or a benzodiazepine in 19 (32.8%).

**Table 2:** Main characteristics of included RCTs according to use of a validated or non-validated definition of delirium

	Validated definition	Non-validated definition	All RCTs
	*N* = 109	*N* = 40	*N* = 149
General characteristics			
Year of publication, *n* (%)			
Before 2010	27 (24.8)	11 (27.5)	38 (25.5)
After 2010	82 (75.2)	29 (72.5)	111 (74.5)
Funding, *n* (%)			
No specific	11 (10.1)	5 (12.5)	16 (10.7)
Public	29 (26.6)	4 (10.0)	33 (22.1)
Private	18 (16.5)	6 (15.0)	24 (16.1)
Public and private	14 (12.8)	3 (7.5)	17 (11.4)
Not reported	37 (33.9)	22 (55.0)	59 (39.6)
Country, *n* (%)			
Canada and USA	34 (31.2)	5 (12.5)	42 (28.2)
Europe	18 (16.5)	14 (35.0)	33 (22.1)
China	19 (17.5)	14 (35.0)	33 (22.1)
Other	23 (21.5)	6 (14.3)	29 (19.5)
Japan	6 (5.5)	1 (2.5)	7 (4.7)
UK	4 (3.7)	2 (5.0)	6 (4.0)
Australia	5 (4.6)	0 (0.0)	5 (3.4)
Center, *n* (%)			
Single-center	59 (54.1)	23 (57.5)	82 (55.0)
Multicenter	39 (33.9)	7 (17.5)	44 (29.5)
Not reported	13 (11.9)	10 (25.0)	23 (15.4)
Sample size, median (*Q*1–*Q*3)	94 (60–142)	80 (54.5–200)	88 (60–164)
Methodological characteristics			
Registration, *n* (%)			
ClinicalTrials.gov	40 (36.7)	3 (7.5)	43 (28.9)
Other	20 (18.3)	4 (10.0)	24 (16.1)
Not reported	49 (45.0)	33 (82.5)	82 (55.0)
Blinding, *n* (%)			
Double-blind	53 (48.6)	19 (47.5)	72 (48.3)
Single-blind (patient)	9 (8.3)	0 (0.0)	9 (6.0)
Open or not reported	47 (43.1)	21 (52.5)	68 (45.7)
Population characteristics			
ICU type, *n* (%)			
Surgical	47 (43.1)	22 (55.0)	69 (46.3)
Medical	8 (7.3)	3 (7.5)	11 (7.4)
Medical and surgical	34 (31.2)	3 (7.5)	37 (24.8)
Not reported	20 (18.3)	12 (30.0)	32 (21.5)
Population type, *n* (%)			
Mechanically ventilated	24 (22.0)	8 (20.0)	32 (21.5)
Older patients	14 (12.8)	1 (2.5)	15 (10.1)
Post-surgery	15 (13.8)	8 (20.0)	23 (15.4)
Post-cardiac surgery	17 (15.6)	11 (27.5)	28 (18.8)
Sepsis	2 (1.8)	0 (0.0)	2 (1.3)
No specific	19 (17.4)	5 (12.5)	24 (16.1)
Main exclusion criteria, *n* (%)			
Dementia	55 (50.5)	5 (12.5)	60 (40.3)
Neurological disorder	41 (37.6)	8 (20.0)	49 (32.9)
Comorbid psychiatric or mood disorder	34 (31.2)	4 (10.0)	38 (25.2)
Chronic antipsychotic use	24 (22.0)	3 (7.5)	27 (18.1)
Alcohol withdrawal	25 (22.9)	4 (10.0)	29 (19.5)
Intervention characteristics			
Type of intervention, *n* (%)			
Pharmacological	78 (71.6)	31 (81.6)	109 (73.1)
Non-pharmacological	29 (26.6)	5 (13.2)	34 (22.8)
Both	2 (1.8)	2 (5.3)	4 (2.7)
No information	0 (0.0)	2 (5.0)	2 (1.5)
Control group, *n* (%)			
Placebo	38 (34.9)	10 (26.3)	48 (32.2)
Usual care	29 (26.6)	7 (18.4)	36 (24.2)
Active pharmacological intervention	41 (37.6)	20 (52.6)	61 (40.9)
Non-pharmacological intervention	1 (0.9)	3 (7.5)	4 (2.7)
Outcome			
Primary outcome, *n* (%)			
Incidence of delirium	43 (39.4)	1 (2.5)	44 (29.5)
Delirium- or coma-free days	7 (6.4)	0 (0.0)	7 (4.7)
Severity of delirium	3 (2.8)	1 (2.5)	4 (2.7)
Number of delirium days	2 (1.8)	0 (0.0)	2 (1.3)
Mortality	3 (2.8)	0 (0.0)	3 (2.0)
ICU length of stay	1 (0.9)	0 (0.0)	1 (0.7)
Other	45 (41.3)	29 (72.5)	88 (59.1)
Not reported	5 (4.6)	9 (22.5)	14 (9.4)
Outcome of delirium evaluated*, *n* (%)			
Incidence of delirium	89 (81.7)	28 (70.0)	117 (78.5)
Number of delirium days	41 (37.6)	2 (5.0)	43 (28.9)
Delirium- or coma-free days	14 (12.8)	0 (0.0)	14 (9.4)
Severity of delirium	8 (7.3)	1 (2.5)	9 (6.0)
Evaluation of risk of bias according to the RoB1, *n* (%)			
Random sequence generation			
High	3 (2.8)	2 (5.0)	5 (3.4)
Low	88 (80.7)	25 (62.5)	113 (75.8)
Unclear	18 (16.5)	13 (32.5)	31 (20.8)
Allocation concealment			
High	10 (9.2)	2 (5.0)	12 (8.1)
Low	69 (63.3)	22 (55.0)	91 (61.1)
Unclear	30 (27.5)	16 (40.0)	46 (30.9)
Blinding of participants and personnel			
High	33 (30.3)	17 (42.5)	50 (33.6)
Low	59 (54.1)	14 (35.0)	73 (49.0)
Unclear	17 (15.6)	9 (22.5)	26 (17.4)
Blinding of outcome assessors			
High	23 (21.1)	16 (40.0)	39 (26.2)
Low	53 (48.6)	13 (32.5)	66 (44.3)
Unclear	33 (30.3)	11 (27.5)	44 (29.5)
Incomplete outcome data			
High	19 (17.6)	5 (12.5)	24 (16.2)
Low	78 (72.2)	22 (55.0)	100 (67.6)
Unclear	11 (10.2)	13 (32.5)	24 (16.2)
Selective outcome reporting			
High	1 (0.9)	0 (0.0)	1 (0.7)
Low	79 (72.5)	29 (72.5)	108 (72.5)
Unclear	29 (26.6)	11 (27.5)	40 (26.8)
Other bias			
High	3 (5.1)	1 (4.3)	4 (4.9)
Low	45 (76.3)	19 (82.6)	64 (78.0)
Unclear	11 (18.6)	3 (13.0)	14 (17.1)

*ICU* intensive care unit, *RoB1* Cochrane Risk of Bias 1 tool

*The total exceeds 149 because some trials evaluated different delirium outcomes

The incidence of delirium was evaluated in 117 (78.5%) trials and was the primary outcome in 44 (29.5%).

### Definition of delirium in included RCTs

**Fig. 1:**
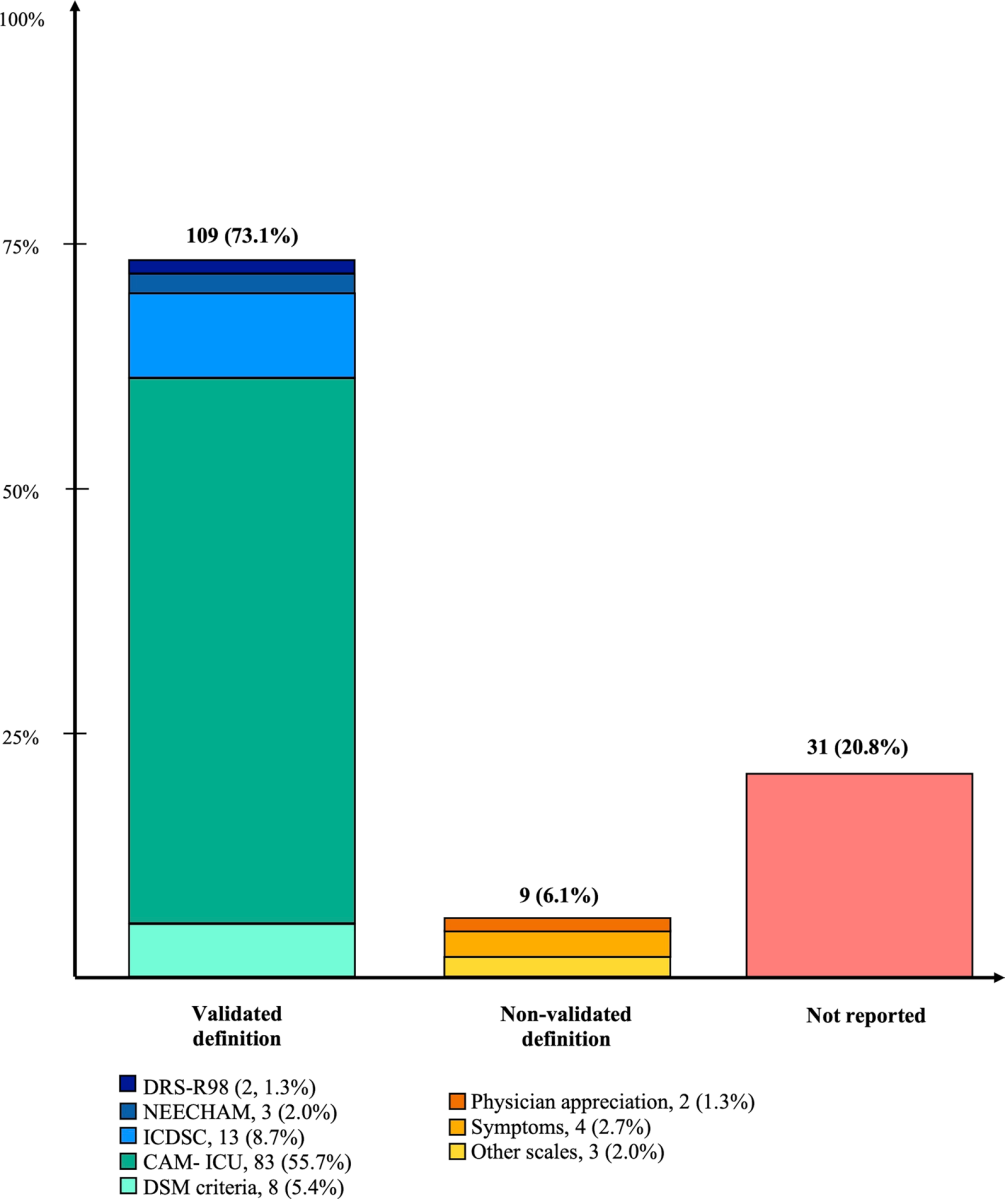
Definition of delirium in included RCTs (*n* = 149). *Legends*: RCT = randomized controlled trial; DSM = diagnostic and statistical manual of mental disorders; CAM-ICU = Confusion Assessment Method for the Intensive Care Unit; ICDSC = Intensive Care Delirium Screening Checklist; DRS-R98 = Delirium Rating Scale Revised-98

Of the 149 RCTs, 109 (73.1%) used a validated definition for delirium and 40 (26.8%) did not. The validated definitions were the DSM criteria in eight RCTs (5.4%), the CAM-ICU in 83 (55.7%), the ICDSC in 13 (8.7%), the NEECHAM scale in three (2.0%) and the DRS-R98 for two (1.3%). Regarding the other trials, three (2.0%) used a non-validated scale (RASS for two, and one did not report the scale used), four (2.7%) a set of symptoms, two (1.3%) left the definition to the physician appreciation and 31 (20.8%) did not report how the delirium was evaluated (Fig. 1).

When extracting the data in RCTs, we found some discrepancies with what was reported in the meta-analyses. Twelve RCTs included in six meta-analyses did not report delirium as an outcome and the authors of the meta-analyses interpreted the delirium as confusion and/or agitation, and/or disorientation, and for six RCTs included in four meta-analyses, which data they considered was not clear.

### Comparison of trial characteristics according to the use of a validated definition of delirium

Reporting the incidence of delirium as a primary outcome was more frequent in RCTs using a validated than non-validated definition (43 [39.4%] vs 1 [2.5%]). Only 7 (17.5%) RCTs using a non-validated definition reported a registration versus 60 (55.0%) using a validated definition. Trials using a non-validated definition were also more likely to be at high or unclear risk of bias for random sequence generation (15 [35.7%] vs 21 [19.3%]), blinding of participants and personnel (26 [65.0%] vs 50 [45.8%]), incomplete outcome data (18 [45.0%] vs 30 [27.8%]) and blinding of outcome assessors (27 [67.5%] vs 56 [51.4%]) (Table 2, Additional file 1: Table S3).

### Meta-epidemiological analyses

#### Primary analysis

**Fig. 2:**
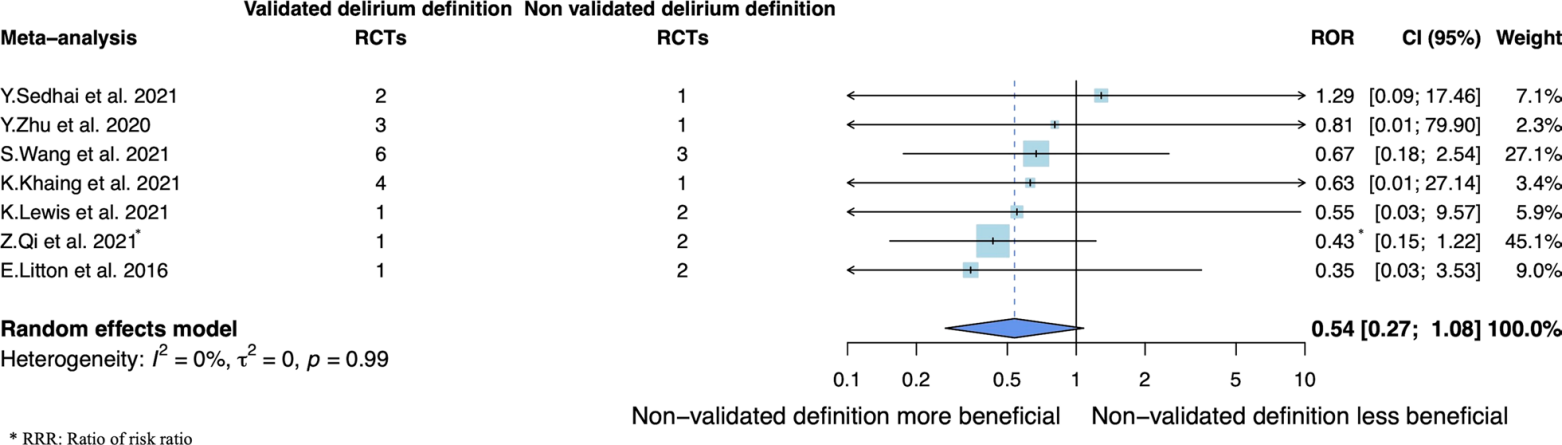
Primary meta-epidemiological analysis using the two-step method: Comparison of intervention effects between trials using a validated definition of delirium (DSM criteria, CAM-ICU, ICDSC, NEECHAM or DRS-R98) and those using a non-validated definition (non-validated scales, set of symptoms, definition left to the physician appreciation or not reported). *Note* An ROR < 1 indicates larger intervention effect estimates for RCTs using a non-validated definition than a validated definition. DSM = diagnostic and statistical manual of mental disorders; CAM-ICU = Confusion Assessment Method for the Intensive Care Unit; ICDSC = Intensive Care Delirium Screening Checklist; DRS-R98 = Delirium Rating Scale Revised-98; RCT = randomized controlled trial; ROR = ratio of odds ratios; CI = confidence interval

Seven meta-analyses (30 RCTs) were included in the primary analysis (Additional file 1: Fig. S2). None of the RCTs meeting inclusion criteria for the meta-epidemiological analysis evaluated delirium with the DRS-R98. We found no significant difference in intervention effects between trials using a validated or a non-validated definition (combined ROR = 0.54, 95% CI 0.27–1.08), with no heterogeneity (*I*^2^ = 0%, *p*_het_ = 0.99, *τ*^2^ = 0) (Fig. 2). Detailed ORs for each meta-analysis according to the use or not of a validated definition are reported in Additional file 1: Fig. S3.

##### Subgroup and sensitivity analyses

The subgroup analysis by type of intervention showed a combined ROR of 0.72 (95% CI 0.26–2.03) for the five meta-analyses evaluating a pharmacological intervention and 0.42 (95% CI 0.16–1.07) for the two meta-analyses evaluating a non-pharmacological intervention (Additional file 1: Fig. S4). The interaction test was non-significant (*p* = 0.44).

For sensitivity analyses, after adjustment for the item of the RoB1 tool and for sample size, RORs were closer to one with large confidence intervals (only three meta-analyses included) (Additional file 1: Fig. S5).

#### Secondary analyses

**Fig. 3:**
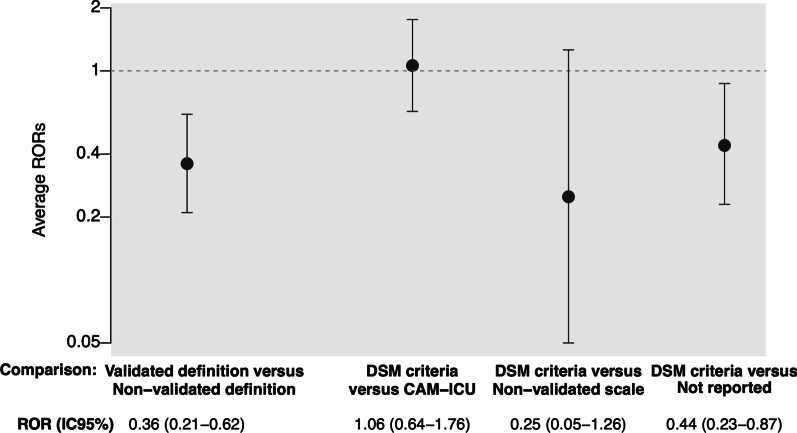
Secondary meta-epidemiological analysis using the multilevel model: Comparison of intervention effects between trials using a validated definition of delirium (DSM criteria, CAM-ICU, ICDSC, NEECHAM or DRS-R98) and those using a non-validated definition (non-validated scales, set of symptoms, definition left to the physician appreciation or not reported), and between trials using the DSM criteria (reference category) and those using the CAM-ICU, a non-validated scale or not reporting the definition used. *Note* This analysis is based on 12 meta-analyses (67 RCTs). For the first ROR, an ROR < 1 indicates larger intervention effect estimates for RCTs using a non-validated definition than a validated definition. For other RORs, an ROR < 1 indicates larger intervention effects for RCTs using the CAM-ICU or a non-validated scale or a definition not reported as compared with the DSM criteria. DSM = diagnostic and statistical manual of mental disorders; CAM-ICU = Confusion Assessment Method for the Intensive Care Unit; ICDSC = Intensive Care Delirium Screening Checklist; DRS-R98 = Delirium Rating Scale Revised-98; RCT = randomized controlled trial; ROR = ratio of odds ratios; CI = confidence interval

The multilevel model analysis included 12 meta-analyses of 67 RCTs and found significantly larger intervention effects in trials using a non-validated versus a validated definition (ROR = 0.36 95% CI 0.21–0.62). We found no significant differences in intervention effects when comparing RCTs using the DSM criteria (reference category) to RCTs using the CAM-ICU (ROR = 1.06, 95% CI 0.64–1.76) or a non-validated scale (ROR = 0.25, 95% CI 0.05–1.26) and found a significantly larger effect for trials not reporting how they defined delirium than those using the DSM criteria (ROR = 0.44, 95% CI 0.23–0.87) (Fig. 3).

## Discussion

In this systematic review of meta-analyses including RCTs assessing prevention or treatment strategies of delirium in ICUs, the definition of delirium was heterogeneous across trials, and one-fifth did not report how they defined delirium. We attempted to assess the impact of this heterogeneity on intervention effect estimates by using a meta-epidemiological approach and found no significant difference between trials using a validated definition and those using a non-validated one in our primary analysis. However, this analysis included few studies and may lack power. The secondary analysis, based on a multilevel model including more studies, found significantly larger intervention effects in trials using a non-validated definition than those using a validated one, which suggests an association between the definition used and intervention effect.

This is the first study evaluating the heterogeneity in the definition of delirium and its association with the intervention effect by a meta-epidemiological approach, the reference method to identify bias [13]. Our sample included mostly recent meta-analyses. The definition of delirium was extracted directly from the RCTs because this information was seldom reported in meta-analyses despite its importance. We classified delirium definitions into different categories specified a priori based on a literature review and considered as a validated definition the DSM criteria because it is the gold standard and CAM-ICU, ICDSC, NEECHAM and DRS-R98 because they have been validated in numerous countries and publications [6, 18–22]. Our classification of validated and non-validated definitions is consistent with the NIDUS list assessment tools for delirium screening [17]. We used two different approaches for the meta-epidemiological analysis and performed sensitivity analyses accounting for important confounding factors.

However, our study has limitations. First, the search strategy might have missed some meta-analyses, but this should not have introduced bias. Second, because meta-analyses were covering the same research area, many RCTs were included in several meta-analyses and had to be removed, which left fewer RCTs available for analysis. Third, it was not reported in included RCTs whether delirium was systematically assessed by research personnel or as part of routine care. Assessment in the context of routine care may lead to under-recognition even when using a validated tool. However, because RCTs are experimental studies with delirium reported as a primary or secondary outcome, we can reasonably assume that it was systematically assessed. Fourth, concerning the meta-epidemiological analysis, only a small number of meta-analyses were included, particularly in the primary analysis, so this analysis may lack power. However, we could not exclude that a difference might exist, especially because the secondary analysis revealed a significant difference. Finally, the development and validation of new tools such as CAM-ICU and ICDSC were concomitant and contributed to an evolution in practices in ICUs with more screening and treatment of delirium. In 2017, the bundle ABCDEF guidelines appeared, representing evidence-based guidelines for physicians to optimize ICU patient care. Management of delirium is a large part of this bundle, which has been increasingly used in the day-to-day care. We tried to collect information on this bundle in trials, but it was seldom reported.

A previous systematic review analyzed the outcomes in RCTs evaluating prevention or treatment strategies of delirium in ICUs [25]. The authors found heterogeneity and multiplicity in outcomes, the most frequent tools used being the CAM-ICU and the ICDSC, which is consistent with our results. They suggested that delirium should be screened regularly with a reliable tool and that a core outcome set be developed to inform delirium research. Another recent systematic review evaluated the heterogeneity in design and analysis of ICU delirium outcome [26]. The authors included RCTs with delirium as primary outcome, evaluated by a validated tool, based on the NIDUS assessment tool list and the DSM criteria, in agreement with our classification. The most frequent tool was the CAM-ICU, which is also consistent with our findings. The authors suggested developing specific methods for statistical analyses and reporting in RCTs of delirium that, if used by most researchers, could improve the quality of clinical trials and the comparison between them. However, they did not raise the issue of the heterogeneity among the definitions of delirium used and included only RCTs using a validated definition. We believe that harmonization in the definition of delirium is the first step to improve the quality of the research on this topic, allowing the research community and physicians to talk about the same thing when considering delirium.

Our study showed heterogeneity in the definition of delirium, with nine different definitions reported, even if CAM-ICU was the most frequently used. The gold standard to define delirium is the DSM criteria, but this evaluation should be made by a psychiatrist. Before the development of tools such as CAM-ICU, ICDSC or NEECHAM, physicians and researchers used various symptoms such as agitation or confusion to define this disorder, thus increasing the heterogeneity in definitions used. One-fifth of trials of delirium did not define delirium. Although *bad reporting does not mean bad methods* [27], the lack of definition limits the interpretation of results including the comparison with other trials. This is why we chose to consider a not-reported definition as non-validated.

Concerning the impact of the heterogeneity in delirium definitions on the intervention effect, the primary meta-epidemiological analysis did not show any significant difference, although the difference was in the direction we expected, which suggests that trials using a non-validated definition may overestimate the intervention effect. However, this analysis lacks power given the small number of studies included. The two-step approach is the most used and the reference method for meta-epidemiological analyses [28], but it is restrictive because it requires including at least one RCT using a validated definition and one using a non-validated definition within each meta-analysis, thus reducing the number of contributing meta-analyses (seven in our study). This constraint does not exist with the multilevel approach, which allows for the inclusion of more meta-analyses. Use of this multilevel model revealed a significant difference in the same direction, which supports the possible existence of larger estimates of intervention effects in trials using a non-validated definition. The secondary analysis with different categories suggests that the difference in intervention effects between trials using a validated and a non-validated definition may be driven by the trials not reporting the definition used. Not reporting the definition of an outcome may reflect a lack of rigor in these trials, which could partly explain the results.

Sensitivity analysis for the primary analysis did not reveal significant differences, but after adjustment for the items of the RoB1, RORs were closer to one. Hence, the non-significantly larger estimates in trials using a non-validated definition may reflect a weaker methodology. In previous meta-epidemiological studies, an inadequate sequence generation, the absence of allocation concealment or lack of blinding was associated with an overestimation of intervention effect [29–32].

Improving how delirium is defined is an important way to limit waste of research [33–35] because it would facilitate the comparison between RCTs, leading to better-quality systematic reviews and meta-analyses on this topic [36, 37]. It may result in a better understanding of this disorder and a better evaluation of the efficacy of therapeutic or preventive interventions. For physicians, it would also improve the diagnosis of delirium, thus resulting in more efficient patient care in ICUs.

The NIDUS proposal listing tools for defining delirium was an important step. However, it includes 34 assessment tools for delirium screening, diagnosis or severity and 5 brief screening tools. Although this catalogue helps clarify the definition of delirium and facilitates the comparisons between trials, there is still a large panel of tools used, which results in heterogeneity with a possible association with the intervention effect. Physicians and researchers should agree on which validated tool should be used to define delirium. The development of a core outcome set may be useful, as suggested by Rose et al. [25].

## Conclusions

This systematic review highlights the heterogeneity in definitions of delirium used in meta-analyses and RCTs assessing its prevention and treatment in ICUs, with one-fifth of trials even not reporting how they evaluated delirium. The primary analysis found no significant difference in intervention effects for RCTs using a validated definition and those using a non-validated one but lacked power given the small number of studies included. The secondary analysis, based on a multilevel model including more RCTs, revealed significantly larger intervention effects for trials using a non-validated than a validated definition. A single and consensual definition of delirium is important for a better evaluation of the interventions to prevent or treat delirium and to improve the quality of research. It may also lead to a better care of patients in everyday practice. The development of a core outcome set on this topic is urgently needed.

## Supplementary Information

**Additional file 1: Table S1:** Prisma Checklist. **Information S1:** Search equation. **Information S2:** Details on the secondary meta-epidemiological analyses conducted. **Table S2:** Detailed characteristics of included meta-analyses. **Table S3:** Main characteristics of the RCTs included according to the tool used to define delirium. **Fig. S1:** Flow chart of the selection of meta-analyses included in the methodological review and RCTs included in the exploration of the heterogeneity in the definition of delirium. **Fig. S2:** Flow chart of the selection of meta-analyses for the primary meta-epidemiological analysis. **Fig. S3:** Comparison of ORs in RCTs using a validated definition of delirium (DSM criteria, CAM-ICU, ICDSC, NEECHAM or DRS-R98) and those using a non-validated (non-validated scales, set of symptoms, definition left to the physician appreciation or not reported) in each meta-analysis. **Fig. S4:** Comparison of intervention effects between trials using a validated definition of delirium (DSM criteria, CAM-ICU, ICDSC, NEECHAM or DRS-R98) and those using a non-validated definition (non-validated scales, set of symptoms, definition left to the physician appreciation or not reported), Subgroup analysis by type of intervention assessed. **Fig. S5:** Comparison of intervention effects between trials using a validated definition of delirium (DSM criteria, CAM-ICU, ICDSC, NEECHAM or DRS-R98) and those using a non-validated definition (non-validated scales, set of symptoms, definition left to the physician appreciation or not reported), Sensitivity analysis adjusted for sample size and each item of the risk of bias tool.

## Data Availability

Available upon request for academic researchers.

## Abbreviations

ICU: Intensive care unit
RCT: Randomized controlled trial
DSM: Diagnostic and Statistical Manual of Mental Disorders
CAM-ICU: Confusion Assessment Method for the Intensive Care Unit
ICDSC: Intensive Care Delirium Screening Checklist
NIDUS: Network for Investigations of Delirium: Unifying Scientists
DRS-R98: Delirium rating scale Revised-98
OR: Odds ratio
ROR: Ratio of odds ratios
CI: Confidence interval
