# Supplementary material for: Heterogeneity in the definition of delirium in ICUs and association with the intervention effect in randomized controlled trials: a meta-epidemiological study
Source: Crit Care. 2023 May 4;27:170. doi: 10.1186/s13054-023-04411-y (PMC10161412; doi:10.1186/s13054-023-04411-y)

**Heterogeneity in the definition of delirium in ICUs and association with intervention effect in randomized controlled trials: a meta-epidemiological study**

Supplemental information - Critical Care

Lucie COLLET^1,2^, MD, Aymeric LANORE*, MD^1,3^, Camille ALATERRE*, MD^2^, Jean-Michel CONSTANTIN, MD, PhD^2^, Guillaume L. MARTIN, MD^1^, Agnès CAILLE, MD, PhD^4^, Arthur JAMES, MD^2^, Agnès DECHARTRES, MD, PhD^1^

* Both authors contributed equally

Correspondance to: Agnès DECHARTRES, MD, PhD. Email: agnes.dechartres@aphp.fr

***Supplemental eTable 1: PRISMA Checklist***

| **Section and Topic** | **Item #** | **Checklist item** | **Location where item is reported** |
| --- | --- | --- | --- |
| **TITLE** | | |  |
| Title | 1 | Identify the report as a systematic review. | p.1 |
| **ABSTRACT** | | |  |
| Abstract | 2 | See the PRISMA 2020 for Abstracts checklist. | NA |
| **INTRODUCTION** | | |  |
| Rationale | 3 | Describe the rationale for the review in the context of existing knowledge. | p.3 |
| Objectives | 4 | Provide an explicit statement of the objective(s) or question(s) the review addresses. | p.3 |
| **METHODS** | | |  |
| Eligibility criteria | 5 | Specify the inclusion and exclusion criteria for the review and how studies were grouped for the syntheses. | p.4 |
| Information sources | 6 | Specify all databases, registers, websites, organisations, reference lists and other sources searched or consulted to identify studies. Specify the date when each source was last searched or consulted. | p.4 |
| Search strategy | 7 | Present the full search strategies for all databases, registers and websites, including any filters and limits used. | SI p.4-5 |
| Selection process | 8 | Specify the methods used to decide whether a study met the inclusion criteria of the review, including how many reviewers screened each record and each report retrieved, whether they worked independently, and if applicable, details of automation tools used in the process. | p.4 |
| Data collection process | 9 | Specify the methods used to collect data from reports, including how many reviewers collected data from each report, whether they worked independently, any processes for obtaining or confirming data from study investigators, and if applicable, details of automation tools used in the process. | p.5-6 |
| Data items | 10a | List and define all outcomes for which data were sought. Specify whether all results that were compatible with each outcome domain in each study were sought (e.g. for all measures, time points, analyses), and if not, the methods used to decide which results to collect. | p.5 |
|  | 10b | List and define all other variables for which data were sought (e.g. participant and intervention characteristics, funding sources). Describe any assumptions made about any missing or unclear information. | p.5 |
| Study risk of bias assessment | 11 | Specify the methods used to assess risk of bias in the included studies, including details of the tool(s) used, how many reviewers assessed each study and whether they worked independently, and if applicable, details of automation tools used in the process. | p.5 |
| Effect measures | 12 | Specify for each outcome the effect measure(s) (e.g. risk ratio, mean difference) used in the synthesis or presentation of results. | p.5 |
| Synthesis methods | 13a | Describe the processes used to decide which studies were eligible for each synthesis (e.g. tabulating the study intervention characteristics and comparing against the planned groups for each synthesis (item #5)). | p.6 |
|  | 13b | Describe any methods required to prepare the data for presentation or synthesis, such as handling of missing summary statistics, or data conversions. | p.5 |
|  | 13c | Describe any methods used to tabulate or visually display results of individual studies and syntheses. | p.5 |
|  | 13d | Describe any methods used to synthesize results and provide a rationale for the choice(s). If meta-analysis was performed, describe the model(s), method(s) to identify the presence and extent of statistical heterogeneity, and software package(s) used. | p.6 |
|  | 13e | Describe any methods used to explore possible causes of heterogeneity among study results (e.g. subgroup analysis, meta-regression). | p.6-7 |
|  | 13f | Describe any sensitivity analyses conducted to assess robustness of the synthesized results. | p.7 |
| Reporting bias assessment | 14 | Describe any methods used to assess risk of bias due to missing results in a synthesis (arising from reporting biases). | p.6 |
| Certainty assessment | 15 | Describe any methods used to assess certainty (or confidence) in the body of evidence for an outcome. | p.6 |
| **RESULTS** | | |  |
| Study selection | 16a | Describe the results of the search and selection process, from the number of records identified in the search to the number of studies included in the review, ideally using a flow diagram. | p.8 |
|  | 16b | Cite studies that might appear to meet the inclusion criteria, but which were excluded, and explain why they were excluded. | p.8 |
| Study characteristics | 17 | Cite each included study and present its characteristics. | p.8 |
| Risk of bias in studies | 18 | Present assessments of risk of bias for each included study. | NA |
| Results of individual studies | 19 | For all outcomes, present, for each study: (a) summary statistics for each group (where appropriate) and (b) an effect estimate and its precision (e.g. confidence/credible interval), ideally using structured tables or plots. | SI p.6-12 |
| Results of syntheses | 20a | For each synthesis, briefly summarise the characteristics and risk of bias among contributing studies. | p.9 |
|  | 20b | Present results of all statistical syntheses conducted. If meta-analysis was done, present for each the summary estimate and its precision (e.g. confidence/credible interval) and measures of statistical heterogeneity. If comparing groups, describe the direction of the effect. | p.9 |
|  | 20c | Present results of all investigations of possible causes of heterogeneity among study results. | p.9 |
|  | 20d | Present results of all sensitivity analyses conducted to assess the robustness of the synthesized results. | p.9 Fig.4 |
| Reporting biases | 21 | Present assessments of risk of bias due to missing results (arising from reporting biases) for each synthesis assessed. | NA |
| Certainty of evidence | 22 | Present assessments of certainty (or confidence) in the body of evidence for each outcome assessed. | p.9 |
| **DISCUSSION** | | |  |
| Discussion | 23a | Provide a general interpretation of the results in the context of other evidence. | p.10 |
|  | 23b | Discuss any limitations of the evidence included in the review. | p.10 |
|  | 23c | Discuss any limitations of the review processes used. | p.10 |
|  | 23d | Discuss implications of the results for practice, policy, and future research. | p.10-11 |
| **OTHER INFORMATION** | | |  |
| Registration and protocol | 24a | Provide registration information for the review, including register name and registration number, or state that the review was not registered. | NA |
|  | 24b | Indicate where the review protocol can be accessed, or state that a protocol was not prepared. | NA |
|  | 24c | Describe and explain any amendments to information provided at registration or in the protocol. | NA |
| Support | 25 | Describe sources of financial or non-financial support for the review, and the role of the funders or sponsors in the review. | p.1 |
| Competing interests | 26 | Declare any competing interests of review authors. | p.1 |
| Availability of data, code and other materials | 27 | Report which of the following are publicly available and where they can be found: template data collection forms; data extracted from included studies; data used for all analyses; analytic code; any other materials used in the review. | NA |

*From:*  Page MJ, McKenzie JE, Bossuyt PM, Boutron I, Hoffmann TC, Mulrow CD, et al. The PRISMA 2020 statement: an updated guideline for reporting systematic reviews. BMJ 2021;372:n71. Doi: 10.1136/bmj.n71

For more information, visit: <http://www.prisma-statement.org/>

***Supplemental Information 1: Search equation***

*Study type: systematic reviews and meta-analysis*

1. “Systematic review” [tiab]
2. “Systematic reviews” [tiab]
3. “Systematic review” [pt]
4. “meta-analysis” [tiab]
5. “meta-analyses” [tiab]
6. “meta analysis” [pt]
7. “overview” [tiab]
8. #1 OR #2 OR #3 OR #4 OR #5 OR #6 OR #7

*Outcome : delirium*

1. “delirium” [tiab]
2. “deliriums” [tiab]
3. delirium [mh]
4. “agitation” [tiab]
5. “agitated” [tiab]
6. “Inattentiveness” [tiab]
7. “Disorientation” [tiab]
8. Hallucinations [mh]
9. “Hallucinations” [tiab]
10. “Hallucination” [tiab]
11. “Psychotic disorder” [tiab]
12. “Psychotic disorders” [tiab]
13. “Psychotic disorders” [mh]
14. “Psychomotor agitation” [mh]
15. “psychosis” [tiab]
16. “delirious” [tiab]
17. “confusion” [tiab]
18. confusion [mh]
19. “confused” [tiab]
20. “Acute confusion” [tiab]
21. “Acute confusional state” [tiab]
22. “Acute brain dysfunction” [tiab]
23. “Altered mental status” [tiab]
24. “Mental status change” [tiab]
25. “encephalopathy” [tiab]
26. “Fluctuating mental status” [tiab]
27. “CAM-ICU” [tiab]
28. “Confusion assessment method” [tiab]
29. “ICDSC” [tiab]
30. “Intensive care delirium screening checklist” [tiab]
31. #9 OR #10 OR #11 OR #12 OR #13 OR #14 OR #15 OR #16 OR #17 OR #18 OR #19 OR #20 OR #21 OR #22 OR #23 OR #24 OR #25 OR #26 OR #27 OR #28 OR #29 OR #30 OR #31 OR #32 OR #33 OR #34 OR #35 OR #36 OR #37 OR #38

*Intensive care unit*

1. “Intensive care” [tiab]
2. “Intensive care units” [mh]
3. “Intensive care units” [tiab]
4. “ICU” OR “ICUs” [tiab]
5. “Critical care” [tiab]
6. “Critical care” [mh]
7. “Critically ill” [tiab]
8. “Critical illness” [mh]
9. “Critical care outcomes” [mh]
10. #40 OR #41 OR #42 OR #43 OR #44 OR #45 OR #46 OR #47 OR #48 OR #49

➔ *Complete equation*

1. #8 AND #39 AND #49

***Supplemental Information 2: Details on the secondary meta-epidemiological analyses conducted***

Regarding the secondary meta-epidemiological analyses, two comparisons were conducted. First, we compared RCTs using a validated definition and those that did not (reference category: validated definition). Then, we compared four definition categories: DSM criteria (reference category), CAM-ICU, non-validated scales and definition not reported.

These secondary meta-epidemiological analyses used a one-step multilevel logistic regression model with random effects to estimate average RORs[1]. We considered the interaction terms between the intervention effect and the definition category as fixed effect. As random effects, we considered a random intercept at the meta-analysis level, a nested (within meta-analysis) random intercept at the trial level and random interaction terms between the intervention effect and the definition categories at the meta-analysis level.

1. Siersma V, Als-Nielsen B, Chen W, et al (2007) Multivariable modelling for meta-epidemiological assessment of the association between trial quality and treatment effects estimated in randomized clinical trials. Stat Med 26:2745–2758. <https://doi.org/10.1002/sim.2752>

*Supplemental eTable 2: Detailed characteristics of included meta-analyses*

| Author - journal | Year | Type of ICU | Population type | Main exclusion criteria | Objective | Type of intervention | Intervention in experimental group | Intervention in control group | Risk of bias assessment tool | Primary outcome | Delirium outcome | Definition of delirium | Number of studies included in the meta-analysis of delirium | Result of MA on delirium | Heterogeneity I^2^  p_het_ |
| --- | --- | --- | --- | --- | --- | --- | --- | --- | --- | --- | --- | --- | --- | --- | --- |
| M. Qin - Journal of Clinical Neuroscience [1] | 2022 | Not reported | Not specific | None | Prevention of delirium | Non- pharmacological | Family intervention | Standard care | RoB 1 | Incidence of delirium | Incidence  Number of days |  | 3 | OR: 0.60 [0.23; 1.59] | I^2^: 24%  p = 0.27 |
| J. Wu - Annals of Palliative Medicine [2] | 2022 | Not reported | Hyperactive brain syndrome | Neurological impairment | Treatment of delirium | Pharmacological | Dexmedetomidine | Haloperidol | RoB 1 | Incidence of delirium | Incidence    Number of days |  | Incidence: 4  Duration: 4 | OR = 0.20 [0.08; 0.46]  SMD: -2.90 [-6.61; 0.80] | I^2^= 0%  p = 0.84  I^2^ =80%  p < 0.01 |
| L. Aitken – Thorax [3] | 2021 | Medical and Surgical | Mechanically ventilated | None | Prevention of delirium | Pharmacological | Light sedation | Deep sedation | RoB 1 | ICU mortality | Incidence |  | 4 | OR: 0.92 [0.65; 1.30] | I^2^: 0%  p = 0.78 |
| K. Khaing - Journal of Psychiatric Research [4] | 2021 | Medical and Surgical | Not specific | None | Prevention of delirium | Pharmacological | Melatonin - Ramelteon | Placebo | RoB 1 | Incidence of delirium | Incidence |  | 5 | OR: 0.50 [0.24; 1.04] | I^2^: 38%  p = 0.17 |
| K. Lewis – Chest [5] | 2021 | Medical and Surgical | Acute respiratory failure with NIV | None | Prevention of delirium | Pharmacological | Dexmedetomidine | Placebo  Other treatments | RoB 2 | Risk of intubation | Incidence |  | 7 | OR: 0.27 [0.16; 0.45] | I^2^: 0%  p = 0.92 |
| Y. Sedhai - Journal of Critical Care [6] | 2021 | Not reported | Not specific | None | Prevention of delirium | Pharmacological | Thiamine | Placebo  Standard care | RoB 1 | Incidence of delirium | Incidence |  | 3 | OR: 0.59 [0.35; 1.00] | I^2:^ 0%  p = 0.38 |
| S. Wang - Journal of Clinical Anesthesia [7] | 2021 | Medical and Surgical | Not specific | None | Prevention and treatment of delirium | Pharmacological | Dexmedetomidine | Placebo  Other treatments | RoB 2 | Incidence of delirium | Incidence  Number of days | Delirium as defined in the original studies | Incidence : 32  Duration: 5 | OR: 0.49 [0.38; 0.64]  SMD: −0.15 [−0.44; 0.14] | I^2^: 64% (p < 0.01)  I^2^: 0%  p = 0.99 |
| S. Liang - Australian Critical Care [8] | 2021 | Medical and Surgical | Not specific | None | Prevention of delirium | Non- pharmacological | Multicomponent or single-component interventions | Standard care | JBI Critical Appraisal Checklists | Incidence and duration of delirium, | Incidence  Number of days |  | Incidence: 7  Duration: 4 | OR: 0.36 [0.26; 0.48]  SMD: −1.12 [−2.16; −0.08] | I^2^: 0%  p = 0.56  I^2^: 88%  p <0.01 |
| S. Zhang - Critical Care Medicine [9] | 2021 | Medical and Surgical | Not specific | None | Prevention of delirium | Non- pharmacological | At least 3 items of ABCDEF Bundle | Not reported | Jadad scale | Incidence of delirium in ICU | Incidence  Number of days | Validated tools (DSM-IV, CAM-ICU, ICDSC) | 3 | OR: 0.92 [0.63; 1.35] | I^2^: 37%  p = 0.20 |
| L. Burry - Intensive Care Medicine [10] | 2021 | Medical and Surgical | Not specific | None | Prevention of delirium | Pharmacological and non-pharmacological | Haloperidol - Dexmedetomidine - Analgesics - Remifentanil - Clonidine - Risperidone - Ramelton - Melatonine - Aripiprazole  IV interruption - IV sedation protocol | Placebo  Standard care  Other treatments | RoB 1 | Delirium occurrence | Incidence  Number of days  Severity |  | Incidence : 20  Incidence environnent intervention : 9  Incidence multicomponent intervention : 6  Duration multicomponent : 3  Duration antipsychotic vs placebo : 5 | OR: 0.73 [0.54; 0.98]  OR: 0.84 [0.49; 1.42]  OR: 0.65 [0.40; 1.05]  SMD: −0.59 [−1.58; 0.39]  SMD: 0.05 [−0.15; 0.25] | I^2^: 53%  p < 0.01  I^2^: 40%  p = 0.11  I^2^: 33%  p = 0.18  I^2^: 82%  p < 0.01  I^2^: 4%  p = 0.38 |
| X.Liu - Minerva Anestesiologica [11] | 2021 | Medical and Surgical | Patient with diagnosed delirium | None | Treatment of delirium | Pharmacological | Dexmedetomidine | Placebo  Other treatments | RoB 1 | Prevalence of delirium and time taken to resolution | Prevalence after treatment  Number of days  Time taken to resolution |  | Prevalence after treatment: 5  Duration: 4  Time to resolution: 5 | OR: 0.35 [0.16; 0.77]  SMD: −0.71 [−2.40; 0.97]  SMD: −0.98 [−2.45; 0.50] | I^2^: 35%  p = 0.19  I^2^: 96%  p < 0.01  I^2^: 95%  p < 0.01 |
| Z. Qi - Australian Critical Care [12] | 2021 | Medical and Surgical | Not specific | None | Prevention of delirium | Non- pharmacological | Nurse-led sedation protocol | Standard care | RoB 1 | Duration of mechanical ventilation | Incidence |  | 3 | RR: 0.52 [0.30; 0.89] | I^2^: 28%  p = 0.25 |
| A. Marra - European Review for Medical and Pharmacological Sciences [13] | 2021 | Medical and Surgical | Not specific | None | Prevention of delirium | Pharmacological | Haloperidol | Placebo  Other treatments | RoB 1 | Incidence of delirium | Incidence  Number of days  Number of delirium and coma free days |  | Incidence: 5  Duration: 4  Delirium and coma free days: 4 | OR: 0.89 [0.60; 1.30]  SMD: −0.06 [−0.43; 0.31]  SMD: −0.05 [−0.18; 0.09] | I^2^: 53%  p = 0.07  I^2^: 37%  p = 0.19  I^2^: 0%  p = 0.52 |
| M.Barbateskovic - Acta Anaesthesiologica Scandinavica [14] | 2020 | Not reported | Patients with delirium at enrollment | None | Treatment of delirium | Pharmacological | Haloperidol | Placebo  Other treatments | RoB 1 | All-cause mortality | Severity |  | 4 | SMD: −0.15 [−0.61; 0.30] | I^2^: 27%  p = 0.25 |
| P.Lin - Heart Lung [15] | 2020 | Medical and Surgical | Not specific | None | Prevention of delirium | Pharmacological | Haloperidol | Placebo | RoB 1 | Incidence of delirium | Incidence |  | 6 | OR = 0.77 [0.52; 1.15] | I^2^ = 55%  p = 0.05 |
| J. Pereira - European Society of Anesthesiology [16] | 2020 | Medical and Surgical | Aged-patients ≥ 60 yo | None | Prevention of delirium | Pharmacological | Dexmedetomidine | Propofol | RoB 1 | Incidence of delirium | Incidence |  | 6 | OR: 0.57 [0.37; 0.87] | I^2^: 0%  p = 0.89 |
| W. Zhou - Experimental and Therapeutic Medicine [17] | 2020 | Medical and Surgical | Not specific | None | Prevention of delirium | Pharmacological | Dexmedetomidine | Midazolam | RoB 1 | Length of ICU stay | Incidence |  | 4 | OR: 0.35 [0.22; 0.55] | I^2^: 0%  p = 0.81 |
| J.Wang - International Journal of Nursing Studies [18] | 2020 | Not reported | Not specific | None | Prevention of delirium | Non- pharmacological | Early mobilization and rehabilitation | Standard care | RoB 1 | Incidence of intensive care unit-acquired weakness | Incidence |  | 4 | OR: 0.27 [0.18; 0.40] | I^2^: 0%  p =0.65 |
| Q. Dong - Pharmacology Research & Perspectives [19] | 2020 | Not reported | Mechanically ventilated | None | Prevention of delirium | Pharmacological | Dexmedetomidine | Midazolam - Lorazepam - Propofol | RoB 1 | Mortality at 30 days | Incidence |  | 3 | OR: 0.43 [0.28; 0.66] | I^2^: 0%  p = 0.38 |
| Z. Chen. - Journal of Clinical Anesthesia [20] | 2020 | Medical and Surgical | Not specific | None | Prevention of delirium | Pharmacological | Haloperidol | Placebo | RoB 1 | Incidence of delirium | Incidence |  | 3 | OR: 0.93 [0.57; 1.51] | I^2^: 66%  p= 0.05 |
| Y. Zhu - Frontiers in neurology [21] | 2020 | Medical and Surgical | Not specific | None | Prevention of delirium | Pharmacological | Melatonin - Ramelteon | Placebo  No treatment  Oxazepam | RoB 1 | Incidence of delirium | Incidence |  | 7 | OR = 0.36 [0.14; 0.92] | I^2^ = 77%  p < 0.01 |
| Y. Zayed - Journal of Critical Care [22] | 2019 | Medical and Surgical | Not specific | None | Prevention of delirium | Pharmacological | Haloperidol | Placebo | RoB 1 | Short-term all-cause mortality | Incidence  Number of delirium and coma free days | CAM-ICU or ICDSC | Incidence: 3  Delirium and coma free days: 5 | OR: 0.93 [0.57; 1.51]  SMD: −0.01 [−0.13; 0.10] | I^2^: 66%  p = 0.05  I^2^: 0%  p = 0.65 |
| L. Burry - Cochrane [23] | 2019 | Medical and Surgical | Confirmed delirium | None | Treatment of delirium | Pharmacological | Dexmedetomidine - Clonidine - Fluoxetine - Haloperidol - Quetiapine - Lorazepam - Rivastigmine - Ketamine - Ramelteon - Morphine - Propofol - Ondansetron - Statins | Placebo  Other treatment  Other non-pharmacological intervention | RoB 1 | Effect of pharmacological interventions for treatment of delirium on duration of delirium | Number of days  Number of delirium and coma free days | DSM criteria (psychiatrists)  CAM-ICU  ICDSC  NEECHAM, Confusion Scale, Delirium Rating Scale (DRS),  DRS-revised-98 | Duration typical AP: 4  Duration atypical AP: 4  Number of coma and delirium free days typical AP: 3 | SMD: −0.06 [−0.31; 0.19]  SMD: −0.24 [−0.56; 0.07]  SMD: 0.74 [−0.30; 1.78] | I^2^: 40%  p = 0.17  I^2^: 59%  p = 0.06  I^2^: 96%  p < 0.01 |
| Q. Zhang - Sleep and Breathing [24] | 2019 | Not reported | Not specific | None | Prevention of delirium | Pharmacological | Melatonin - Ramelteon | Placebo | RoB 1 | Incidence of delirium | Incidence |  | 3 | OR: 0.24 [0.11; 0.54] | I^2^: 19%  p = 0.29 |
| K. Ng – Anesthesia [25] | 2019 | Medical and Surgical | Not specific | None | Prevention of delirium | Pharmacological | Dexmedetomidine | Placebo | RoB 1 | Incidence of delirium and agitation | Incidence |  | 8 | OR: 0.37 [0.26; 0.51] | I^2^: 0%  p = 0.94 |
| L.Bannon - Intensive Care Medicine [26] | 2019 | Medical and Surgical | Not specific | None | Prevention of delirium | Non- pharmacological | Multicomponent non-pharmacological intervention | Standard care | RoB 1 | Incidence and duration of delirium | Incidence    Number of days |  | 4 | OR: 0.35 [0.08; 1.63] | I^2^: 73%  p = 0.01 |
| C.Wang - Intensive and Critical Care Nursing [27] | 2019 | Medical and Surgical | Not specific | None | Prevention of delirium | Pharmacological | Analgesia - analgosedation - no sedation | Standard care  Hyptonic based sedation | RoB 1 | 28-days or hospital mortality | Incidence |  | 4 | OR: 0.83 [0.31; 2.20] | I^2^: 71%  p = 0.02 |
| J. Flükiger - Annals of Intensive Care [28] | 2018 | Medical and Surgical | Not specific | None | Prevention and treatment of delirium | Pharmacological | Dexmedetomidine | Placebo  Other treatments | RoB 1 | Incidence of delirium | Incidence |  | Placebo-controlled: 10  Standard sedative controlled: 15 | OR: 0.39 [0.25; 0.62]  OR: 0.51 [0.34; 0.77] | I^2^: 48%  p = 0.05  I^2^: 55%  p < 0.01 |
| J. Kang - Journal of Critical Care [29] | 2018 | Medical and Surgical | Not specific | None | Prevention of delirium | Non- pharmacological | Multicomponent non-pharmacological intervention | Standard care | RoB 1 | Incidence of delirium | Incidence |  | 10 | OR: 0.78 [0.56; 1.07] | I^2^: 49%  p = 0.04 |
| H. Fan - Iran J Public Health [30] | 2017 | Surgical | Post-surgery | None | Prevention of delirium | Pharmacological | Dexmedetomidine | Placebo  Propofol | RoB 1 | Prevalence of delirium | Incidence |  | 7 | OR = 0.44 [0.22; 0.86] | I^2^ = 65%  p < 0.01 |
| A. Nassar - Revista Brasileira de Terapia Intensiva [31] | 2016 | Medical and Surgical | Not specific | None | Prevention of delirium | Non- pharmacological | Predefined sedation scale target with daily sedative infusion interruption | Mild target sedation protocol | RoB 1 | Mortality in the ICU | Incidence |  | 3 | OR: 0.69 [0.37; 1.30] | I^2^: 43%  p = 0.18 |
| E. Litton - Critical Care Medicine [32] | 2016 | Medical and Surgical | Not specific | None | Prevention of delirium | Non- pharmacological | Earplugs | Eye mask - soothing music | RoB 1 | Incidence of delirium | Incidence |  | 3 | OR: 0.92 [0.42; 2.01] | I^2^: 0%  p = 0.41 |
| JM Constantin - Anaesth Crit Care Pain Med [33] | 2016 | Medical and Surgical | Not specific | Patients who underwent cardiac surgery | Prevention of delirium | Pharmacological | Dexmedetomidine | Propofol - Midazolam - Lorazepam - Haloperidol | Not evaluated | ICU length of stay | Incidence |  | 8 | OR: 0.66 [0.35; 1.22] | I^2^: 68%  p < 0.01 |
| J. Porhomayon - Journal of Cardiovascular and Thoracic Research [34] | 2015 | Medical and Surgical | Not specific | Neuro-ICU or stroke | Prevention of delirium | Pharmacological | Low sedation | Standard care  Heavy sedation (Midazolam) | Not reported | Incidence of cognitive dysfunction | Incidence | Validated standard method | 4 | OR: 0.85 [0.44; 1.64] | I^2^: 31%  p = 0.22 |
| R. Serafim - Journal of Critical Care [35] | 2015 | Medical and Surgical | Not specific | None | Prevention or Treatment of delirium | Pharmacological | Rivastigmine - Haloperidol - Ziprasidone - Quetiapine - Dexmedetomidine | Placebo  Other treatments | RoB 1 | Incidence of delirium  Delirium resolution | Incidence    Number of delirium and coma free days |  | 3 | SMD: 0.14 [−0.17; 0.45] | I^2^: 47%  p = 0.15 |
| K. Chen – Cochrane [36] | 2015 | Medical and Surgical | Mechanically ventilated | Sedation for less than 24 hours | Prevention of delirium | Pharmacological | Dexmedetomidine | Standard care  Other treatments | RoB 1 | Duration of mechanical ventilation | Incidence | Any diagnostic criteria | 7 | OR = 0.67 [0.34; 1.32] | I^2^ = 74%  p < 0.01 |
| N. Al Qadheeb - Critical Care Medicine [37] | 2014 | Not reported | Not specific | None | Treatment of delirium | Pharmacological and non-pharmacological | Quetiapine - Dexmedetomidine - Clonidine - Rivastigmine - Risperidone - Ziprasidone  Early physiotherapy - Spontaneous awakening trial - Spontaneous breathing trial | Placebo  Standard care  No treatment  Other treatments | RoB 1 | Duration of delirium | Number of days | Validated technique CAM-ICU, ICDSC, DSM-IV criteria by a psychiatrist or neurologist | 17 | SMD: −0.64 [ −1.14; −0.13] | I^2^: 66%  p < 0.01 |
| L. Burry – Cochrane [38] | 2014 | Medical and Surgical | Mechanically ventilated | None | Prevention of delirium | Non-pharmacological | Daily sedation interruption with other sedation strategies | Standard care | RoB 1 | Duration of invasive ventilation | Incidence |  | 3 | OR = 1.00 [0.75; 1.33] | I^2^ = 0%  p = 0.54 |
| L.Pasin - Journal of Cardiothoracic and Vascular Anesthesia [39] | 2014 | Medical and Surgical | Not specific | None | Prevention of delirium | Pharmacological | Dexmedetomidine | Placebo  Other treatments | RoB 1 | Rate of delirium | Incidence |  | 14 | OR: 0.57 [0.34; 0.95] | I^2^: 75%  p <0.01 |
| Z. Xia - Journal of Surgical Resaerch [40] | 2013 | Medical and Surgical | Not specific | Dexmedetomidine used for anesthesia | Prevention of delirium | Pharmacological | Dexmedetomidine | Propofol | RoB 1 | Length of ICU stay | Incidence |  | 3 | OR = 0.58 [0.31; 1.09] | I^2^= 0% (p = 0.51) |
| J. Tan - Intensive Care Medicine [41] | 2010 | Medical and Surgical | Not specific | None | Prevention of delirium | Pharmacological | Dexmedetomidine | Placebo  Other treatments | RoB 1 | Length of ICU stay, length of mechanical ventilation, bradycardia/ hypotension | Incidence |  | 8 | OR: 0.67 [0.32; 1.37] | I^2^: 72%  p < 0.01 |

References of included meta-analyses

1. Qin M, Gao Y, Guo S, et al (2022) Family intervention for delirium for patients in the intensive care unit: A systematic meta-analysis. J Clin Neurosci 96:114–119. https://doi.org/10.1016/j.jocn.2021.11.011

2. Wu J, Li B, Ma K, et al (2022) A systematic review and meta-analysis of the clinical efficacy of the intravenous injection of dexmedetomidine in ICU patients with hyperactive brain syndrome. Ann Palliat Med 11:299–308. https://doi.org/10.21037/apm-21-3762

3. Aitken LM, Kydonaki K, Blackwood B, et al (2021) Inconsistent relationship between depth of sedation and intensive care outcome: systematic review and meta-analysis. Thorax 76:1089–1098. https://doi.org/10.1136/thoraxjnl-2020-216098

4. Khaing K, Nair BR (2021) Melatonin for delirium prevention in hospitalized patients: A systematic review and meta-analysis. J Psychiatr Res 133:181–190. https://doi.org/10.1016/j.jpsychires.2020.12.020

5. Lewis K, Piticaru J, Chaudhuri D, et al (2021) Safety and Efficacy of Dexmedetomidine in Acutely Ill Adults Requiring Noninvasive Ventilation: A Systematic Review and Meta-analysis of Randomized Trials. Chest 159:2274–2288. https://doi.org/10.1016/j.chest.2020.12.052

6. Sedhai YR, Shrestha DB, Budhathoki P, et al (2021) Effect of thiamine supplementation in critically ill patients: A systematic review and meta-analysis. J Crit Care 65:104–115. https://doi.org/10.1016/j.jcrc.2021.05.016

7. Wang S, Hong Y, Li S, et al (2021) Effect of dexmedetomidine on delirium during sedation in adult patients in intensive care units: A systematic review and meta-analysis. J Clin Anesth 69:110157. https://doi.org/10.1016/j.jclinane.2020.110157

8. Liang S, Chau JPC, Lo SHS, et al (2021) Effects of nonpharmacological delirium-prevention interventions on critically ill patients’ clinical, psychological, and family outcomes: A systematic review and meta-analysis. Aust Crit Care 34:378–387. https://doi.org/10.1016/j.aucc.2020.10.004

9. Zhang S, Han Y, Xiao Q, et al (2021) Effectiveness of Bundle Interventions on ICU Delirium: A Meta-Analysis. Crit Care Med 49:335–346. https://doi.org/10.1097/CCM.0000000000004773

10. Burry LD, Cheng W, Williamson DR, et al (2021) Pharmacological and non-pharmacological interventions to prevent delirium in critically ill patients: a systematic review and network meta-analysis. Intensive Care Med 47:943–960. https://doi.org/10.1007/s00134-021-06490-3

11. Liu X, Xiong J, Tang Y, et al (2021) Role of dexmedetomidine in the treatment of delirium in critically ill patients: a systematic review and meta-analysis. Minerva Anestesiol 87:65–76. https://doi.org/10.23736/S0375-9393.20.14492-4

12. Qi Z, Yang S, Qu J, et al (2021) Effects of nurse-led sedation protocols on mechanically ventilated intensive care adults: A systematic review and meta-analysis. Aust Crit Care 34:278–286. https://doi.org/10.1016/j.aucc.2020.07.013

13. Marra A, Vargas M, Buonanno P, et al (2021) Haloperidol for preventing delirium in ICU patients: a systematic review and meta-analysis. Eur Rev Med Pharmacol Sci 25:1582–1591. https://doi.org/10.26355/eurrev_202102_24868

14. Barbateskovic M, Krauss SR, Collet MO, et al (2020) Haloperidol for the treatment of delirium in critically ill patients: A systematic review with meta-analysis and Trial Sequential Analysis. Acta Anaesthesiol Scand 64:254–266. https://doi.org/10.1111/aas.13501

15. Lin P, Zhang J, Shi F, Liang Z-A (2020) Can haloperidol prophylaxis reduce the incidence of delirium in critically ill patients in intensive care units? A systematic review and meta-analysis. Heart Lung 49:265–272. https://doi.org/10.1016/j.hrtlng.2020.01.010

16. Pereira JV, Sanjanwala RM, Mohammed MK, et al (2020) Dexmedetomidine versus propofol sedation in reducing delirium among older adults in the ICU: A systematic review and meta-analysis. Eur J Anaesthesiol 37:121–131. https://doi.org/10.1097/EJA.0000000000001131

17. Zhou W-J, Liu M, Fan X-P (2021) Differences in efficacy and safety of midazolam vs. dexmedetomidine in critically ill patients: A meta-analysis of randomized controlled trial. Exp Ther Med 21:156. https://doi.org/10.3892/etm.2020.9297

18. Wang J, Ren D, Liu Y, et al (2020) Effects of early mobilization on the prognosis of critically ill patients: A systematic review and meta-analysis. Int J Nurs Stud 110:103708. https://doi.org/10.1016/j.ijnurstu.2020.103708

19. Dong Q, Li C, Xiao F, Xie Y (2020) Efficacy and safety of dexmedetomidine in patients receiving mechanical ventilation: Evidence from randomized controlled trials. Pharmacol Res Perspect 8:e00658. https://doi.org/10.1002/prp2.658

20. Chen Z, Chen R, Zheng D, et al (2020) Efficacy and safety of haloperidol for delirium prevention in adult patients: An updated meta-analysis with trial sequential analysis of randomized controlled trials. J Clin Anesth 61:109623. https://doi.org/10.1016/j.jclinane.2019.09.017

21. Zhu Y, Jiang Z, Huang H, et al (2020) Assessment of Melatonergics in Prevention of Delirium: A Systematic Review and Meta-Analysis. Front Neurol 11:198. https://doi.org/10.3389/fneur.2020.00198

22. Zayed Y, Barbarawi M, Kheiri B, et al (2019) Haloperidol for the management of delirium in adult intensive care unit patients: A systematic review and meta-analysis of randomized controlled trials. J Crit Care 50:280–286. https://doi.org/10.1016/j.jcrc.2019.01.009

23. Burry L, Hutton B, Williamson D, et al (2019) Pharmacological interventions for the treatment of delirium in critically ill adults. Cochrane Database of Systematic Reviews. https://doi.org/10.1002/14651858.CD011749.pub2

24. Zhang Q, Gao F, Zhang S, et al (2019) Prophylactic use of exogenous melatonin and melatonin receptor agonists to improve sleep and delirium in the intensive care units: a systematic review and meta-analysis of randomized controlled trials. Sleep Breath 23:1059–1070. https://doi.org/10.1007/s11325-019-01831-5

25. Ng KT, Shubash CJ, Chong JS (2019) The effect of dexmedetomidine on delirium and agitation in patients in intensive care: systematic review and meta-analysis with trial sequential analysis. Anaesthesia 74:380–392. https://doi.org/10.1111/anae.14472

26. Bannon L, McGaughey J, Verghis R, et al (2019) The effectiveness of non-pharmacological interventions in reducing the incidence and duration of delirium in critically ill patients: a systematic review and meta-analysis. Intensive Care Med 45:1–12. https://doi.org/10.1007/s00134-018-5452-x

27. Wang C-T, Mao Y, Zhao L, Ma B (2019) The impact of analgosedation on mortality and delirium in critically ill patients: A systematic review and meta-analysis. Intensive Crit Care Nurs 54:7–14. https://doi.org/10.1016/j.iccn.2019.06.004

28. Flükiger J, Hollinger A, Speich B, et al (2018) Dexmedetomidine in prevention and treatment of postoperative and intensive care unit delirium: a systematic review and meta-analysis. Ann Intensive Care 8:92. https://doi.org/10.1186/s13613-018-0437-z

29. Kang J, Lee M, Ko H, et al (2018) Effect of nonpharmacological interventions for the prevention of delirium in the intensive care unit: A systematic review and meta-analysis. J Crit Care 48:372–384. https://doi.org/10.1016/j.jcrc.2018.09.032

30. Fan H, Zhao Y, Sun M, et al (2017) Dexmedetomidine Based Sedation for Post-surgery Critically Ill Adults: A Meta-analysis of Randomized Controlled Trials. Iran J Public Health 46:1611–1622

31. Nassar APJ, Park M (2016) Sedation protocols versus daily sedation interruption: a systematic review and meta-analysis. Rev Bras Ter Intensiva 28:444–451. https://doi.org/10.5935/0103-507X.20160078

32. Litton E, Carnegie V, Elliott R, Webb SAR (2016) The Efficacy of Earplugs as a Sleep Hygiene Strategy for Reducing Delirium in the ICU: A Systematic Review and Meta-Analysis. Crit Care Med 44:992–999. https://doi.org/10.1097/CCM.0000000000001557

33. Constantin J-M, Momon A, Mantz J, et al (2016) Efficacy and safety of sedation with dexmedetomidine in critical care patients: a meta-analysis of randomized controlled trials. Anaesth Crit Care Pain Med 35:7–15. https://doi.org/10.1016/j.accpm.2015.06.012

34. Porhomayon J, Joude P, Adlparvar G, et al (2015) The Impact of High Versus Low Sedation Dosing Strategy on Cognitive Dysfunction in Survivors of Intensive Care Units: A Systematic Review and Meta-Analysis. J Cardiovasc Thorac Res 7:43–48. https://doi.org/10.15171/jcvtr.2015.10

35. Serafim RB, Bozza FA, Soares M, et al (2015) Pharmacologic prevention and treatment of delirium in intensive care patients: A systematic review. J Crit Care 30:799–807. https://doi.org/10.1016/j.jcrc.2015.04.005

36. Chen K, Lu Z, Xin Y, et al (2015) Alpha‐2 agonists for long‐term sedation during mechanical ventilation in critically ill patients. Cochrane Database of Systematic Reviews. https://doi.org/10.1002/14651858.CD010269.pub2

37. Al-Qadheeb NS, Balk EM, Fraser GL, et al (2014) Randomized ICU trials do not demonstrate an association between interventions that reduce delirium duration and short-term mortality: a systematic review and meta-analysis. Crit Care Med 42:1442–1454. https://doi.org/10.1097/CCM.0000000000000224

38. Burry L, Rose L, McCullagh I, et al (2014) Daily sedation interruption versus no daily sedation interruption for critically ill adult patients requiring invasive mechanical ventilation. Cochrane Database of Systematic Reviews. https://doi.org/10.1002/14651858.CD009176.pub2

39. Pasin L, Landoni G, Nardelli P, et al (2014) Dexmedetomidine reduces the risk of delirium, agitation and confusion in critically Ill patients: a meta-analysis of randomized controlled trials. J Cardiothorac Vasc Anesth 28:1459–1466. https://doi.org/10.1053/j.jvca.2014.03.010

40. Xia Z-Q, Chen S-Q, Yao X, et al (2013) Clinical benefits of dexmedetomidine versus propofol in adult intensive care unit patients: a meta-analysis of randomized clinical trials. J Surg Res 185:833–843. https://doi.org/10.1016/j.jss.2013.06.062

41. Tan JA, Ho KM (2010) Use of dexmedetomidine as a sedative and analgesic agent in critically ill adult patients: a meta-analysis. Intensive Care Med 36:926–939. https://doi.org/10.1007/s00134-010-1877-6

*Supplemental eTable 3: Main characteristics of the RCTs included according to the tool used to define delirium*

|  | Validated definition | | | | | Non-validated definition | | | |
| --- | --- | --- | --- | --- | --- | --- | --- | --- | --- |
|  | **DSM criteria** | **CAM-ICU** | **ICDSC** | **NEECHAM** | **DRS-R98** | **Other scale** | **Set of symptoms** | **Clinician appreciation** | **Not reported** |
|  | **N = 8** | **N = 83** | **N = 13** | **N = 3** | **N = 2** | **N = 3** | **N = 4** | **N = 2** | **N = 31** |
| General characteristics |  |  |  |  |  |  |  |  |  |
| Year of publication, n (%) |  |  |  |  |  |  |  |  |  |
| Before 2010 | 6 (75.0) | 16 (19.3) | 2 (15.4) | 1 (33.3) | 2 (100.0) | 0 (0.0) | 1 (25.0) | 0 (0.0) | 10 (32.3) |
| After 2010 | 2 (25.0) | 67 (80.7) | 11 (84.6) | 2 (66.7) | 0 (0.0) | 3 (10.0) | 3 (75.0) | 2 (100.0) | 21 (67.7) |
| Funding, n (%) |  |  |  |  |  |  |  |  |  |
| No specific | 0 (0.0) | 9 (10.8) | 1 (7.7) | 1 (33.3) | 0 (0.0) | 2 (66.7) | 2 (50.0) | 0 (0.0) | 1 (3.2) |
| Public | 2 (25.0) | 22 (26.5) | 3 (23.1) | 0 (0.0) | 2 (100.0) | 0 (0.0) | 0 (0.0) | 0 (0.0) | 4 (12.9) |
| Private | 0 (0.0) | 14 (16.9) | 4 (30.8) | 0 (0.0) | 0 (0.0) | 0 (0.0) | 2 (50.0) | 0 (0.0) | 4 (12.9) |
| Public and Private | 1 (12.5) | 12 (14.5) | 1 (7.7) | 0 (0.0) | 0 (0.0) | 0 (0.0) | 0 (0.0) | 0 (0.0) | 3 (9.7) |
| Not reported | 5 (62.5) | 26 (31.3) | 4 (30.8) | 2 (66.7) | 0 (0.0) | 1 (33.3) | 0 (0.0) | 2 (100.0) | 19 (61.3) |
| Country, n (%) |  |  |  |  |  |  |  |  |  |
| Canada and United-States | 3 (37.5) | 23 (27.7) | 6 (46.2) | 0 (0.0) | 2 (100.0) | 0 (0.0) | 0 (0.0) | 0 (0.0) | 5 (16.1) |
| Europe | 3 (37.5) | 15 (18.1) | 0 (0.0) | 1 (33.3) | 0 (0.0) | 1 (33.3) | 2 (50.0) | 0 (0.0) | 11 (35.5) |
| China | 0 (0.0) | 18 (22.0) | 1 (7.7) | 0 (0.0) | 0 (0.0) | 0 (0.0) | 0 (0.0) | 1 (50.0) | 13 (41.9) |
| Other | 0 (0.0) | 14 (16.7) | 4 (30.8) | 0 (0.0) | 0 (0.0) | 2 (66.6) | 1 (25.0) | 1 (50.0) | 0 (0.0) |
| Japan | 2 (25.0) | 2 (2.4) | 0 (0.0) | 2 (66.7) | 0 (0.0) | 0 (0.0) | 0 (0.0) | 0 (0.0) | 1 (3.2) |
| United-Kingdom | 0 (0.0) | 4 (4.8) | 0 (0.0) | 0 (0.0) | 0 (0.0) | 0 (0.0) | 2 (50.0) | 0 (0.0) | 0 (0.0) |
| Australia | 0 (0.0) | 4 (4.8) | 1 (7.7) | 0 (0.0) | 0 (0.0) | 0 (0.0) | 0 (0.0) | 0 (0.0) | 1 (3.0) |
| Center, n (%) |  |  |  |  |  |  |  |  |  |
| Single-center | 6 (75.0) | 45 (50.6) | 6 (46.2) | 3 (100.0) | 2 (100.0) | 3 (100.0) | 1 (25.0) | 2 (100.0) | 17 (54.8) |
| Multicenter | 2 (25.0) | 30 (36.1) | 5 (38.5) | 0 (0.0) | 0 (0.0) | 0 (0.0) | 2 (50.0) | 0 (0.0) | 5 (16.1) |
| Not reported | 0 (0.0) | 11 (13.3) | 2 (15.4) | 0 (0.0) | 0 (0.0) | 0 (0.0) | 1 (25.0) | 0 (0.0) | 9 (29.0) |
| Methodological characteristics |  |  |  |  |  |  |  |  |  |
| Registration, n (%) |  |  |  |  |  |  |  |  |  |
| ClinicalTrials | 1 (12.5) | 33 (39.8) | 6 (46.2) | 0 (0.0) | 0 (0.0) | 0 (0.0) | 2 (50.0) | 0 (0.0) | 1 (3.2) |
| Other | 2 (25.0) | 17 (20.5) | 0 (0.0) | 1 (33.3) | 0 (0.0) | 0 (0.0) | 0 (0.0) | 1 (50.0) | 3 (9.7) |
| Not reported | 5 (62.5) | 33 (39.8) | 7 (53.8) | 2 (66.7) | 2 (100.0) | 3 (100.0) | 2 (50.0) | 1 (50.0) | 27 (87.1) |
| Blinding, n (%) |  |  |  |  |  |  |  |  |  |
| Double-blind | 2 (25.0) | 42 (50.6) | 7 (53.8) | 0 (0.0) | 2 (100.0) | 3 (100.0) | 4 (100.0) | 1 (50.0) | 11 (35.5) |
| Single-blind (patient) | 1 (12.5) | 8 (9.6) | 0 (0.0) | 0 (0.0) | 0 (0.0) | 0 (0.0) | 0 (0.0) | 0 (0.0) | 0 (0.0) |
| Open or Not reported | 5 (62.5) | 33 (39.8) | 6 (46.2) | 3 (100.0) | 0 (0.0) | 0 (0.0) | 0 (0.0) | 1 (50.0) | 20 (64.5) |
| Population characteristics |  |  |  |  |  |  |  |  |  |
| ICUs type, n (%) |  |  |  |  |  |  |  |  |  |
| Medical and Surgical | 1 (12.5) | 25 (30.1) | 7 (53.8) | 1 (33.3) | 0 (0.0) | 0 (0.0) | 2 (50.0) | 0 (0.0) | 1 (3.2) |
| Surgical | 6 (75.0) | 37 (44.6) | 2 (15.4) | 2 (66.7) | 0 (0.0) | 2 (66.6) | 2 (50.0) | 1 (50.0) | 17 (54.8) |
| Medical | 1 (12.5) | 6 (7.2) | 0 (0.0) | 0 (0.0) | 1 (50.0) | 0 (0.0) | 0 (0.0) | 0 (0.0) | 3 (9.7) |
| Not reported | 0 (0.0) | 15 (18.1) | 4 (30.8) | 0 (0.0) | 1 (50.0) | 1 (33.3) | 0 (0.0) | 1 (50.0) | 10 (32.3) |
| Population type, n (%) |  |  |  |  |  |  |  |  |  |
| Mechanically ventilated | 1 (12.5) | 19 (22.9) | 4 (30.8) | 0 (0.0) | 0 (0.0) | 0 (0.0) | 2 (50.0) | 0 (0.0) | 6 (19.4) |
| Older-patients | 2 (25.0) | 11 (13.3) | 1 (7.7) | 0 (0.0) | 0 (0.0) | 0 (0.0) | 1 (25.0) | 0 (0.0) | 0 (0.0) |
| Post-surgery | 1 (12.5) | 11 (13.3) | 1 (7.7) | 2 (66.7) | 0 (0.0) | 0 (0.0) | 0 (0.0) | 0 (0.0) | 8 (25.8) |
| Post-cardiac surgery | 4 (50.0) | 13 (15.7) | 0 (0.0) | 0 (0.0) | 0 (0.0) | 2 (66.6) | 1 (25.0) | 1 (50.0) | 7 (22.6) |
| Sepsis | 0 (0.0) | 2 (2.4) | 0 (0.0) | 0 (0.0) | 0 (0.0) | 0 (0.0) | 0 (0.0) | 0 (0.0) | 0 (0.0) |
| No specific | 0 (0.0) | 15 (18.1) | 3 (23.1) | 1 (33.3) | 0 (0.0) | 0 (0.0) | 0 (0.0) | 0 (0.0) | 5 (16.1) |
| Main exclusion criteria, n (%) |  |  |  |  |  |  |  |  |  |
| Dementia | 3 (37.5) | 45 (54.2) | 5 (38.5) | 2 (66.7) | 0 (0.0) | 0 (0.0) | 2 (50.0) | 0 (0.0) | 3 (9.7) |
| Neurological disorder | 2 (25.0) | 33 (39.8) | 4 (30.8) | 0 (0.0) | 2 (100.0) | 0 (0.0) | 3 (75.0) | 0 (0.0) | 5 (16.1) |
| Comorbid psychiatric or mood disorder | 4 (50.0) | 27 (32.5) | 1 (7.7) | 0 (0.0) | 2 (100.0) | 1 (33.3) | 1 (25.0) | 0 (0.0) | 2 (6.5) |
| Chronic antipsychotic use | 4 (50.0) | 16 (19.3) | 2 (15.4) | 0 (0.0) | 2 (100.0) | 0 (0.0) | 1 (25.0) | 1 (50.0) | 1 (3.2) |
| Alcohol withdrawal | 4 (50.0) | 13 (15.7) | 6 (46.2) | 0 (0.0) | 2 (100.0) | 0 (0.0) | 1 (25.0) | 0 (0.0) | 3 (9.7) |
| Intervention characteristics |  |  |  |  |  |  |  |  |  |
| Type of intervention, n (%) |  |  |  |  |  |  |  |  |  |
| Pharmacological | 7 (87.5) | 58 (69.9) | 11 (84.6) | 0 (0.0) | 2 (100.0) | 3 (100.0) | 4 (100.0) | 2 (100.0) | 22 (71.0) |
| Non-pharmacological | 0 (0.0) | 24 (28.9) | 2 (15.4) | 3 (100.0) | 0 (0.0) | 0 (0.0) | 0 (0.0) | 0 (0.0) | 5 (16.1) |
| Both | 1 (12.5) | 1 (1.2) | 0 (0.0) | 0 (0.0) | 0 (0.0) | 0 (0.0) | 0 (0.0) | 0 (0.0) | 2 (6.5) |
| No information | 0 (0.0) | 0 (0.0) | 0 (0.0) | 0 (0.0) | 0 (0.0) | 0 (0.0) | 0 (0.0) | 0 (0.0) | 2 (6.5) |
| Control group, n (%) |  |  |  |  |  |  |  |  |  |
| Placebo | 4 (50.0) | 29 (34.9) | 5 (38.5) | 0 (0.0) | 0 (0.0) | 1 (33.3) | 0 (0.0) | 0 (0.0) | 9 (31.0) |
| Usual care | 1 (12.5) | 24 (28.9) | 1 (7.7) | 3 (100.0) | 0 (0.0) | 0 (0.0) | 0 (0.0) | 0 (0.0) | 7 (24.1) |
| Active pharmacological intervention | 3 (37.5) | 29 (34.9) | 7 (53.8) | 0 (0.0) | 2 (100.0) | 2 (66.6) | 4 (100.0) | 2 (100.0) | 12 (41.4) |
| Non-pharmacological intervention | 0 (0.0) | 1 (1.2) | 0 (0.0) | 0 (0.0) | 0 (0.0) | 0 (0.0) | 0 (0.0) | 0 (0.0) | 3 (9.7) |
| Outcome |  |  |  |  |  |  |  |  |  |
| Primary outcome, n (%) |  |  |  |  |  |  |  |  |  |
| Incidence of delirium | 7 (87.5) | 33 (39.8) | 2 (15.4) | 1 (33.3) | 0 (0.0) | 0 (0.0) | 1 (25.0) | 0 (0.0) | 0 (0.0) |
| Delirium or coma free days | 0 (0.0) | 7 (8.4) | 0 (0.0) | 0 (0.0) | 0 (0.0) | 0 (0.0) | 0 (0.0) | 0 (0.0) | 0 (0.0) |
| Severity of delirium | 0 (0.0) | 1 (1.2) | 0 (0.0) | 0 (0.0) | 2 (100.0) | 1 (33.3) | 0 (0.0) | 0 (0.0) | 0 (0.0) |
| Number of delirium days | 0 (0.0) | 2 (2.4) | 0 (0.0) | 0 (0.0) | 0 (0.0) | 0 (0.0) | 0 (0.0) | 0 (0.0) | 0 (0.0) |
| Mortality | 0 (0.0) | 3 (3.6) | 0 (0.0) | 0 (0.0) | 0 (0.0) | 0 (0.0) | 0 (0.0) | 0 (0.0) | 0 (0.0) |
| ICU length of stay | 0 (0.0) | 1 (1.2) | 0 (0.0) | 0 (0.0) | 0 (0.0) | 0 (0.0) | 0 (0.0) | 0 (0.0) | 0 (0.0) |
| Other | 1 (12.5) | 32 (38.6) | 9 (69.2) | 2 (66.6) | 0 (0.0) | 2 (66.6) | 3 (75.0) | 2 (100.0) | 22 (71.0) |
| Not reported | 0 (0.0) | 3 (3.6) | 2 (15.4) | 0 (0.0) | 0 (0.0) | 0 (0.0) | 0 (0.0) | 0 (0.0) | 9 (29.0) |
| Outcome of delirium evaluated*, n (%) |  |  |  |  |  |  |  |  |  |
| Incidence of delirium | 8 (100.0) | 71 (85.5) | 7 (53.8) | 3 (100.0) | 0 (0.0) | 2 (66.6) | 4 (100.0) | 2 (100.0) | 20 (64.5) |
| Number of delirium days | 4 (50.0) | 31 (37.3) | 6 (46.2) | 0 (0.0) | 0 (0.0) | 0 (0.0) | 0 (0.0) | 0 (0.0) | 2 (6.5) |
| Delirium or coma free days | 0 (0.0) | 14 (16.9) | 0 (0.0) | 0 (0.0) | 0 (0.0) | 0 (0.0) | 0 (0.0) | 0 (0.0) | 0 (0.0) |
| Severity of delirium | 3 (37.5) | 3 (3.6) | 0 (0.0) | 0 (0.0) | 2 (100.0) | 1 (33.3) | 0 (0.0) | 0 (0.0) | 0 (0.0) |
| Evaluation of risk of bias according to the RoB 1, n (%) |  |  |  |  |  |  |  |  |  |
| Random sequence generation |  |  |  |  |  |  |  |  |  |
| High | 0 (0.0) | 3 (3.6) | 0 (0.0) | 0 (0.0) | 0 (0.0) | 1 (33.3) | 0 (0.0) | 0 (0.0) | 1 (3.2) |
| Low | 8 (100.0) | 66 (79.5) | 10 (76.9) | 2 (66.7) | 2 (100.0) | 1 (33.3) | 4 (100.0) | 1 (50.0) | 19 (61.3) |
| Unclear | 0 (0.0) | 14 (16.9) | 3 (23.1) | 1 (33.3) | 0 (0.0) | 1 (33.3) | 0 (0.0) | 1 (50.0) | 11 (35.5) |
| Allocation concealment |  |  |  |  |  |  |  |  |  |
| High | 1 (12.5) | 9 (10.8) | 0 (0.0) | 0 (0.0) | 0 (0.0) | 0 (0.0) | 0 (0.0) | 0 (0.0) | 2 (6.5) |
| Low | 5 (62.5) | 53 (63.9) | 9 (69.2) | 2 (66.7) | 0 (0.0) | 1 (33.3) | 4 (100.0) | 1 (50.0) | 16 (51.6) |
| Unclear | 2 (25.0) | 21 (25.3) | 4 (30.8) | 1 (33.3) | 2 (100.0) | 2 (66.6) | 0 (0.0) | 1 (50.0) | 13 (41.9) |
| Blinding of participants and personnel |  |  |  |  |  |  |  |  |  |
| High | 5 (62.5) | 21 (25.3) | 5 (38.5) | 2 (66.7) | 0 (0.0) | 0 (0.0) | 0 (0.0) | 0 (0.0) | 17 (54.8) |
| Low | 2 (25.0) | 48 (57.8) | 6 (46.2) | 1 (33.3) | 2 (100.0) | 1 (33.3) | 4 (100.0) | 1 (50.0) | 8 (25.8) |
| Unclear | 1 (12.5) | 14 (16.9) | 2 (15.4) | 0 (0.0) | 0 (0.0) | 2 (66.6) | 0 (0.0) | 1 (50.0) | 6 (19.4) |
| Blinding of outcome assessors |  |  |  |  |  |  |  |  |  |
| High | 3 (37.5) | 15 (18.1) | 3 (23.1) | 2 (66.7) | 0 (0.0) | 0 (0.0) | 0 (0.0) | 0 (0.0) | 16 (51.6) |
| Low | 3 (37.5) | 41 (49.4) | 6 (46.2) | 1 (33.3) | 2 (100.0) | 1 (33.3) | 3 (75.0) | 1 (50.0) | 8 (25.8) |
| Unclear | 2 (25.0) | 27 (32.5) | 4 (30.8) | 0 (0.0) | 0 (0.0) | 2 (66.6) | 1 (25.0) | 1 (50.0) | 7 (22.6) |
| Incomplete outcome data |  |  |  |  |  |  |  |  |  |
| High | 2 (28.6) | 14 (16.9) | 3 (23.1) | 0 (0.0) | 0 (0.0) | 0 (0.0) | 1 (25.0) | 1 (50.0) | 3 (9.7) |
| Low | 4 (57.1) | 61 (73.5) | 9 (69.2) | 2 (66.7) | 2 (100.0) | 2 (66.6) | 0 (0.0) | 1 (50.0) | 19 (61.3) |
| Unclear | 1 (14.3) | 8 (9.6) | 1 (7.7) | 1 (33.3) | 0 (0.0) | 1 (33.3) | 3 (75.0) | 0 (0.0) | 9 (29.0) |
| Selective outcome reporting |  |  |  |  |  |  |  |  |  |
| High | 1 (12.5) | 0 (0.0) | 0 (0.0) | 0 (0.0) | 0 (0.0) | 0 (0.0) | 0 (0.0) | 0 (0.0) | 0 (0.0) |
| Low | 6 (75.0) | 62 (74.7) | 9 (69.2) | 2 (66.7) | 0 (0.0) | 1 (33.3) | 3 (75.0) | 1 (50.0) | 24 (77.4) |
| Unclear | 1 (12.5) | 21 (25.3) | 4 (30.8) | 1 (33.3) | 2 (100.0) | 2 (66.6) | 1 (25.0) | 1 (50.0) | 7 (22.6) |
| Other bias |  |  |  |  |  |  |  |  |  |
| High | 0 (0.0) | 1 (2.2) | 2 (22.2) | 0 (0.0) | NR | 0 (0.0) | 0 (0.0) | 0 (0.0) | 1 (5.3) |
| Low | 4 (100.0) | 35 (77.8) | 5 (55.6) | 1 (100.0) | NR | 0 (0.0) | 1 (100.0) | 1 (50.0) | 17 (89.5) |
| Unclear | 0 (0.0) | 9 (20.0) | 2 (22.2) | 0 (0.0) | NR | 1 (100.0) | 0 (0.0) | 1 (50.0) | 1 (5.3) |

* The total exceeds 149 as some trials evaluated different delirium outcomes

**Supplemental eFig. 1: Flow chart of the selection of meta-analyses included in the methodological review and RCTs included in the exploration of the heterogeneity in the definition of delirium**

Records identified from:

MEDLINE (n = 514)

Cochrane Database of Systematic Review (n = 41)

Identification

Records removed *before screening*:

Duplicate records removed

(n = 15 )

Records screened

(n = 540)

Screening

Records excluded

(n = 435)

Reports sought for retrieval

(n = 105)

Reports not retrieved

(n = 2)

Reports assessed for eligibility

(n = 103)

Reports excluded:

No MA (n = 23)

No ICU subgroup (n = 23)

MA including less than 3 studies (n = 9)

Outcome delirium not available (n = 4)

No RCT included (n = 3)

Included

**Meta-analyses included in methodological review**

**(n = 41)**

**(n = 300 studies)**

Studies excluded:

No RCTs (n = 12)

No ICU (n = 3)

Pediatric population (n = 1)

284 RCTs in ICU in adult populations

**149 unique RCTs**

RCTs excluded:

Duplicates as defined (n = 135)

**Supplemental eFig. 2: Flow chart of the selection of meta-analyses for the primary meta-epidemiological analysis**

Meta-analyses included in methodological review

(n = 41)

Meta-analyses not evaluating incidence of delirium

(n = 3)

Meta-analyses included in methodological review evaluating the incidence of delirium

(n = 38)

Group control different of placebo, standard care or no treatment:

(n = 17)

Meta-analysis comparing an intervention to placebo, standard care or no treatment with more than 3 RCTs included

(n = 21)

Meta-analyses with no heterogeneity in the definition of delirium:

(n = 11)

Meta-analysis comparing an intervention to placebo, standard care or no treatment with heterogeneity in the definition of delirium

(n = 10)

Meta-analysis having the same research objective and including 3 or more studies in common:

(n = 3)

**Meta-analyses included in the main meta-epidemiological analysis**

**(n = 7)**

**(n = 30 RCTs)**

**Supplemental eFig. 3: Comparison of ORs in RCTs using a validated definition of delirium (DSM criteria, CAM-ICU, ICDSC, NEECHAM or DRS-R98) and those using a non-validated (non-validated scales, set of symptoms, definition left to the physician appreciation or not reported) in each meta-analysis**

***
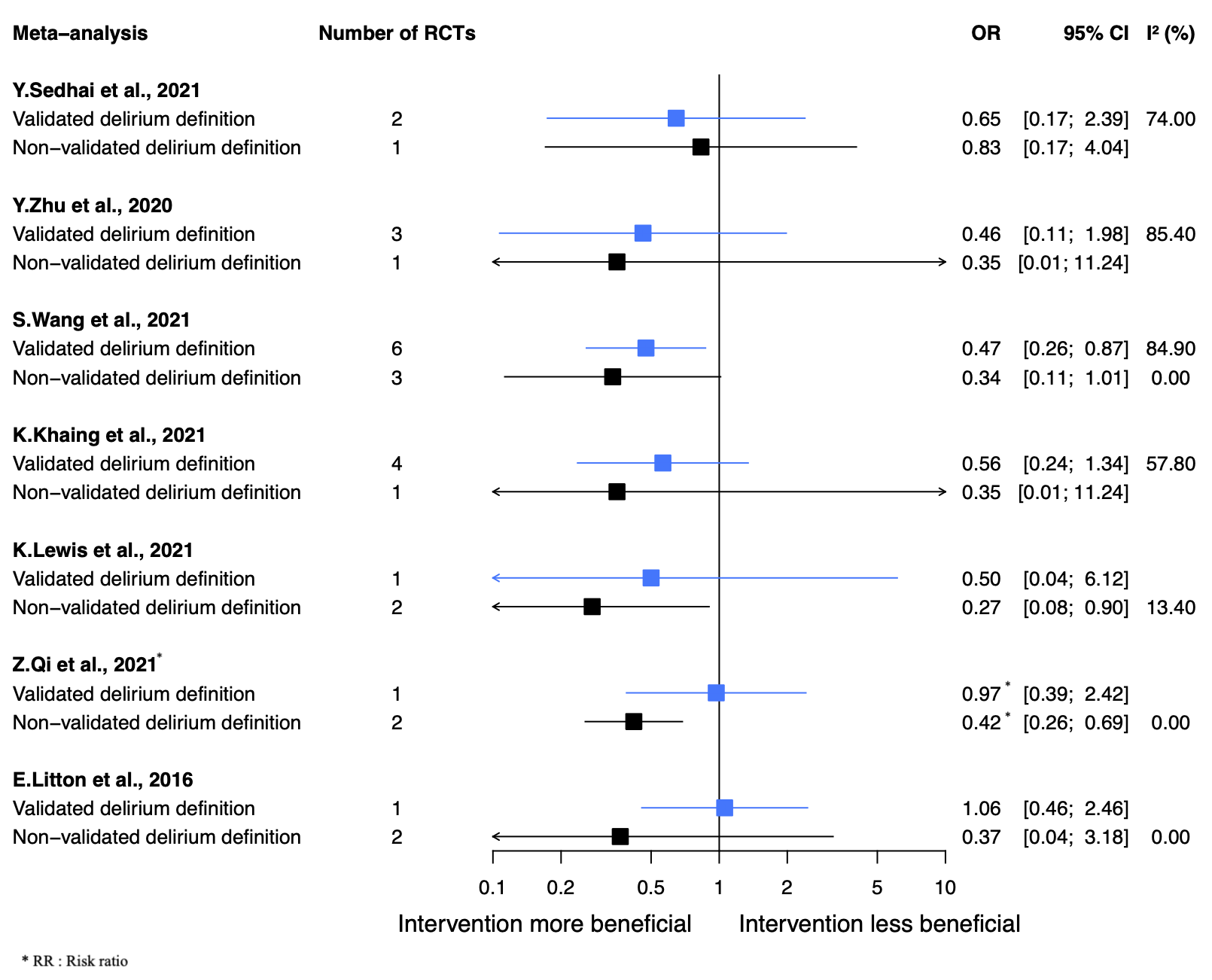
***

**Supplemental eFig. 4: Comparison of intervention effects between trials using a validated definition of delirium (DSM criteria, CAM-ICU, ICDSC, NEECHAM or DRS-R98) and those using a non-validated definition (non-validated scales, set of symptoms, definition left to the physician appreciation or not reported), Subgroup analysis by type of intervention assessed**

***
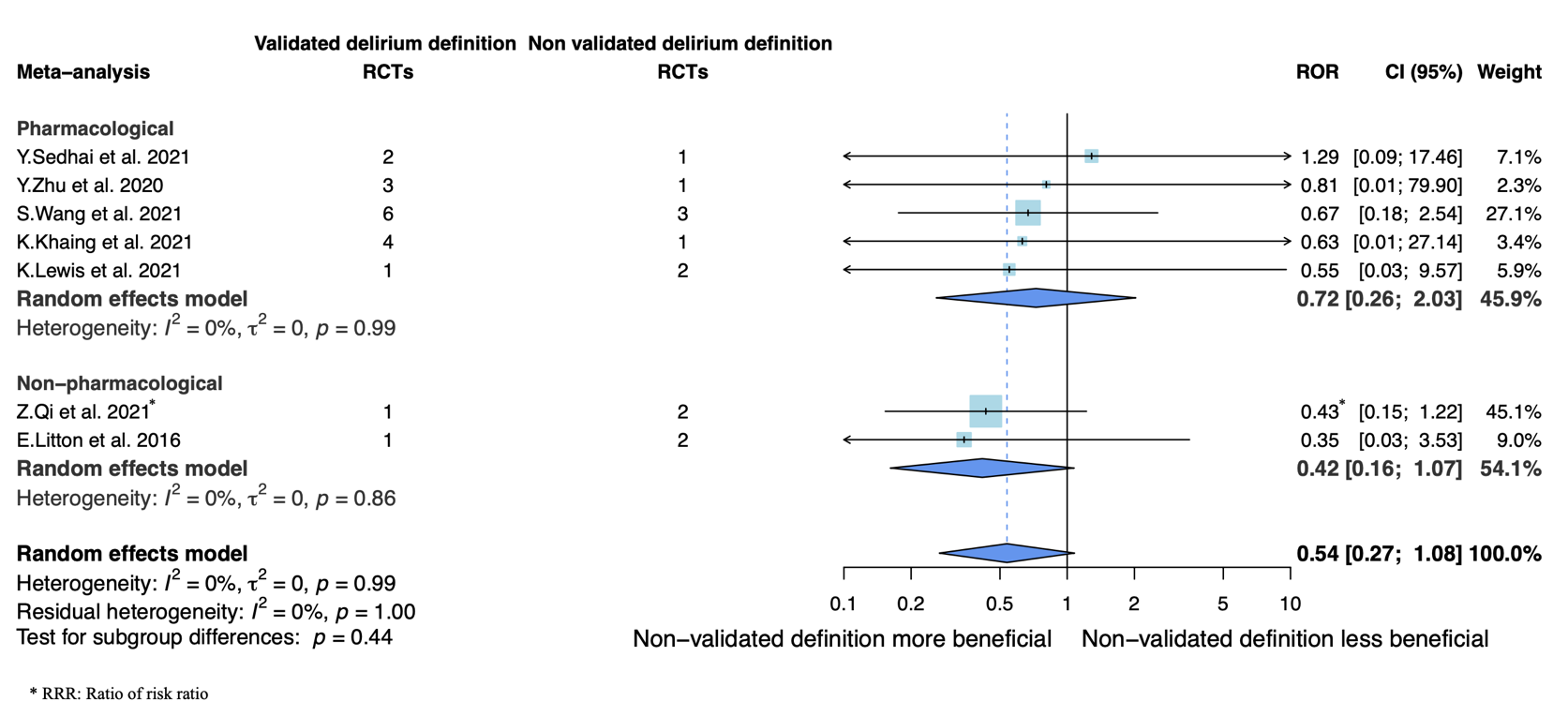
***

**Supplemental eFig. 5: Comparison of intervention effects between trials using a validated definition of delirium (DSM criteria, CAM-ICU, ICDSC, NEECHAM or DRS-R98) and those using a non-validated definition (non-validated scales, set of symptoms, definition left to the physician appreciation or not reported), Sensitivity analysis adjusted for sample size and each item of the risk of bias tool**


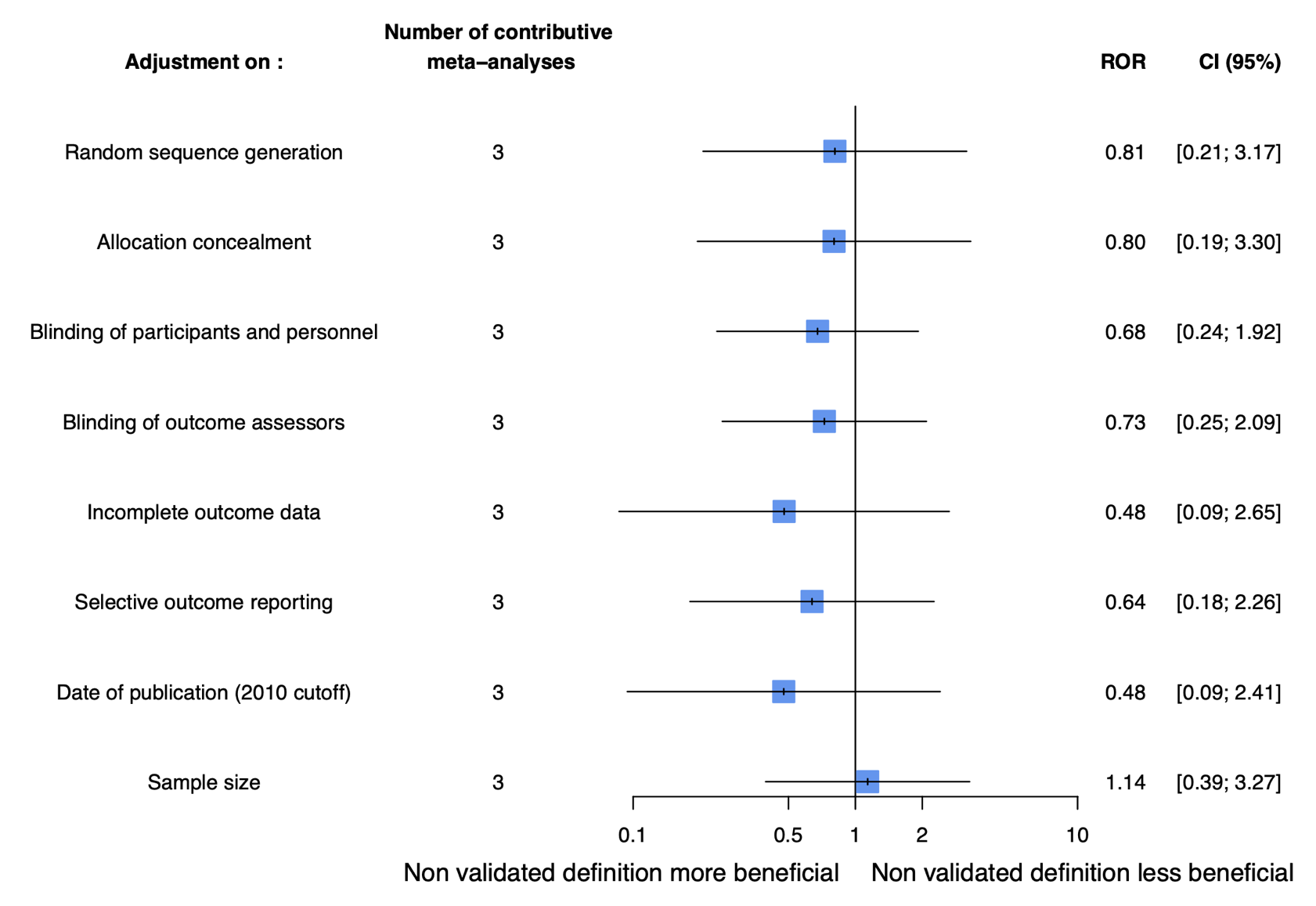

Supplement: Supplementary file 1 — Additional file 1: Table S1: Prisma Checklist. Information S1: Search equation. Information S2: Details on the secondary meta-epidemiological analyses conducted. Table S2: Detailed characteristics of included meta-analyses. Table S3: Main characteristics of the RCTs included according to the tool used to define delirium. Fig. S1: Flow chart of the selection of meta-analyses included in the methodological review and RCTs included in the exploration of the heterogeneity in the definition of delirium. Fig. S2: Flow chart of the selection of meta-analyses for the primary meta-epidemiological analysis. Fig. S3: Comparison of ORs in RCTs using a validated definition of delirium (DSM criteria, CAM-ICU, ICDSC, NEECHAM or DRS-R98) and those using a non-validated (non-validated scales, set of symptoms, definition left to the physician appreciation or not reported) in each meta-analysis. Fig. S4: Comparison of intervention effects between trials using a validated definition of delirium (DSM criteria, CAM-ICU, ICDSC, NEECHAM or DRS-R98) and those using a non-validated definition (non-validated scales, set of symptoms, definition left to the physician appreciation or not reported), Subgroup analysis by type of intervention assessed. Fig. S5: Comparison of intervention effects between trials using a validated definition of delirium (DSM criteria, CAM-ICU, ICDSC, NEECHAM or DRS-R98) and those using a non-validated definition (non-validated scales, set of symptoms, definition left to the physician appreciation or not reported), Sensitivity analysis adjusted for sample size and each item of the risk of bias tool. [file 13054_2023_4411_MOESM1_ESM.docx]
